# Chronic neuroinflammation during aging leads to cholinergic neurodegeneration in the mouse medial septum

**DOI:** 10.1186/s12974-023-02897-5

**Published:** 2023-10-13

**Authors:** Rashmi Gamage, Ilaria Rossetti, Garry Niedermayer, Gerald Münch, Yossi Buskila, Erika Gyengesi

**Affiliations:** 1 School of Medicine, Western Sydney University, Penrith, NSW 2751 Australia; 2 School of Science, Western Sydney University, Penrith, NSW 2751 Australia

**Keywords:** Neuroinflammation, Healthy aging, Microglia, Cholinergic system, Animal-mouse, Stereology, 3D reconstruction

## Abstract

**Background:**

Low-grade, chronic inflammation in the central nervous system characterized by glial reactivity is one of the major hallmarks for aging-related neurodegenerative diseases like Alzheimer’s disease (AD). The basal forebrain cholinergic neurons (BFCN) provide the primary source of cholinergic innervation of the human cerebral cortex and may be differentially vulnerable in various neurodegenerative diseases. However, the impact of chronic neuroinflammation on the cholinergic function is still unclear.

**Methods:**

To gain further insight into age-related cholinergic decline, we investigated the cumulative effects of aging and chronic neuroinflammation on the structure and function of the septal cholinergic neurons in transgenic mice expressing interleukin-6 under the GFAP promoter (GFAP-IL6), which maintains a constant level of gliosis. Immunohistochemistry combined with unbiased stereology, single cell 3D morphology analysis and in vitro whole cell patch-clamp measurements were used to validate the structural and functional changes of BFCN and their microglial environment in the medial septum.

**Results:**

Stereological estimation of MS microglia number displayed significant increase across all three age groups, while a significant decrease in cholinergic cell number in the adult and aged groups in GFAP-IL6 mice compared to control. Moreover, we observed age-dependent alterations in the electrophysiological properties of cholinergic neurons and an increased excitability profile in the adult GFAP-IL6 group due to chronic neuroinflammation. These results complimented the significant decrease in hippocampal pyramidal spine density seen with aging and neuroinflammation.

**Conclusions:**

We provide evidence of the significant impact of both aging and chronic glial activation on the cholinergic and microglial numbers and morphology in the MS, and alterations in the passive and active electrophysiological membrane properties of septal cholinergic neurons, resulting in cholinergic dysfunction, as seen in AD. Our results indicate that aging combined with gliosis is sufficient to cause cholinergic disruptions in the brain, as seen in dementias.

**Supplementary Information:**

The online version contains supplementary material available at 10.1186/s12974-023-02897-5.

## Introduction

Aging is the most prevalent and unmodifiable risk factor in Alzheimer’s disease (AD), and age-related deterioration of both the peripheral and central components of the immune system increase our vulnerability to infections and diseases [1, 2]. Neuroinflammation is a reactive process of the central nervous system (CNS) which strives to retain homeostasis, by protecting against extrinsic deleterious factors. Neuroinflammation is a major emerging contributor to neurodegenerative diseases like AD [3]. It is now well accepted that chronic inflammation in the CNS affects β-amyloid (Aβ) accumulation, tau phosphorylation and synaptic and neuronal loss associated cognitive deficits in AD [4, 5].

The primary component of the CNS immune system are the microglial cells, which independently activate, in response to foreign or harmful pathogens to maintain the cellular microenvironment [1]. However, when microglia maintain a chronic state of activation due to sustained stimulation, it could lead to a toxic microenvironment for the surrounding neuronal cells [6, 7]. Subsequently, degenerating neurons would release various pro-inflammatory cytokines that in turn exacerbate the inflammatory response of microglia, potentiating the neurodegenerative cycle [8]. It is well established that aging is a contributor to microglial senescence and substantial phenotypic changes in them could lead to associated neurodegenerative events [9–12].

The basal forebrain (BF) cholinergic system is composed of structures including the medial septum (MS), ventral pallidum (VP), vertical and horizontal diagonal band nuclei (VDB, HDB), substantia innominata/extended amygdala (SI/EA), and peripallidal regions [9, 13–16]. While the BF consist of a heterogeneous mixture of neuron types, a prominent feature that determines its boundaries is the collection of aggregated and non-aggregated large projection neurons containing choline acetyl transferase (ChAT), innervating the cerebral cortex, including the cingulate cortex, subiculum, entorhinal cortex, hippocampal formation, and the amygdala [9, 15–17]. The main source of cholinergic innervation to the entire extent of the dorsal and ventral hippocampus is delivered via the septo-hippocampal pathway, in which cholinergic projections from the MS/VDB passing through via the fimbria-fornix [18, 19], and are termed Ch1 and Ch2 subdivisions, respectively [20]. In fact, nearly 65%, of septal projections to the hippocampus are cholinergic, and they primarily target the dendrites of CA1 pyramidal neurons [21–24].

The BF cholinergic neurons (BFCN) play an important role in regulating the sleep–wake cycle, information processing related to cognitive function, and are directly involved in regulating circuits of attention and memory, including social interaction and social recognition memory, throughout the lifespan [17, 25–27]. The reciprocal connections between the MS/VDB and the hippocampus are important in the generation and maintenance of the hippocampal theta and gamma oscillatory activity which evidently supplement information processing, formation of new memories, voluntary movement, rapid eye movement (REM) sleep, and arousal [15, 21, 28, 29].

Previous studies suggested that the BFCNs are vulnerable to normal aging and their degeneration coincide with cognitive decline exhibited by AD patients [25, 27]. Additionally, the resultant loss of cholinergic inputs to the hippocampus from the BF can contribute to AD memory impairments, as the hippocampus is a key structure in learning and memory [18, 19, 23, 30]. Due to their complex axonal arborization, which requires a great expenditure of energy for growth, maintenance, and repair, the BFCNs are highly vulnerable to perturbed energy homeostasis, oxidative stress, and neuroinflammation, which are all increased during aging [31]. However, the primary underlying mechanism leading to cholinergic loss is still not clearly defined, but studies suggests that chronic inflammatory processes may contribute to the pathology and should be investigated as a novel therapeutic target [2, 27, 32]. Recently, our group showed that acute neuroinflammation (via peripheral injections of LPS) led to a differential impact on their membrane properties, increasing the excitability profile of cholinergic neurons in adult mice (9–12 months) without affecting the other age groups [33]. Thus, in this study we focused on the impact of aging and chronic neuroinflammation on the vulnerability of cholinergic neurons which might increase their susceptibility to degeneration. We investigated changes to the septal microglia and cholinergic cell population during normal aging and pathological aging, using the glial fibrillary acidic protein-interleukin 6 (GFAP-IL6) transgenic mouse model [34]. We have previously reported hippocampal and cerebellar pathology related to progressive neurodegeneration including; neuronal loss and atrophy, chronic activated microglia and astrocytes, increased expression of inflammatory mediators, and age-dependent motor and cognitive impairment in GFAP-IL6 mice [35–39]. Therefore, we further extended our investigation to identify differences in hippocampal CA1 pyramidal cell spine density with aging and neuroinflammation, and to see if these changes could be related to our current findings on septal cholinergic cell population.

## Materials and methods

### Animals

The experiments were performed on transgenic heterozygous GFAP-IL6 or the ChAT-eGFAP/GFAP-IL6 mice [aged 3–4 months (young); 10–12 months (adult); 18–24 months (old)] and age-matched C57BL/6/ChAT^(BAC)^-eGFP mice of mixed genders (*n = *15 per cohort). The ChAT^(BAC)^-eGFP [40] transgenic mice (strain 007902) were obtained from Jax laboratories and bred in house. The GFAP-IL6 [34] transgenic mice (strain C57BL/6) were a generous gift from Prof Iain Campbell (University of Sydney).

The pro-inflammatory mediator IL-6 has been implicated in AD as a link to aggravate cognitive impairments and disease associated pathology [4, 41, 42]. The GFAP-IL6 transgenic mouse model was generated as a chronic neuroinflammation model to investigate cytokine signalling within the CNS [34, 42]. In this model, astroglia express IL-6 gene under the transcriptional control of GFAP promoter, ensuing brain-specific overexpression of IL-6 [34]. This transgenic mouse model replicates neuropathological changes of human neurodegenerative diseases including AD. Our group and others have reported, astrocytosis, microgliosis, angiogenesis, neurodegeneration, progressive learning deficits, cerebellar volume loss, and motor deficits in the GFAP-IL6 mice [35–39, 43].

The ChAT^(BAC)^-eGFP mice were created by adding a bacterial artificial chromosome (BAC) clone into the ChAT locus, in which enhanced green fluorescent protein (eGFP) is inserted into exon 3 at the ChAT initiation codon, strongly and selectively express eGFP in all of the CNS and PNS cholinergic neurons, as well as in the non-neuronal cells [40]. There was almost perfect overlap of both ChAT^(BAC)^-eGFP and Gt-a-ChAT-labelled neurons in the entire expansion of the BF including the MS, validating the use of the ChAT^(BAC)^-eGFP transgenic strain or C57BL/6 stain as the age-matched control for this study [44].

The GFAP-IL6 animal genotype was confirmed by PCR analysis of DNA collected from ear notches, as standardized. All animals weighing 20–30 g were housed in individually ventilated cages (IVC), in the animal facility of the School of Medicine, Western Sydney University under a temperature-controlled environment, with a normal 12 h/12 h light/dark cycle at 23 °C, 60 ± 10% humidity, and provided with standard mouse chow and water ad libitum. The housing included basic enrichments including domes, nesting material (crinkle nest), and a pipe tube (polyvinyl chloride).

To assess the electrophysiological properties of the cholinergic neurons in the MS, we crossbred the GFAP-IL6 and the ChAT(BAC)-eGFP mouse models described above, creating the ChAT-eGFAP/GFAP-IL6 line. The genotype was confirmed by PCR analysis of DNA collected from ear notches, as standardized. The experiments were performed on transgenic ChAT-eGFAP/GFAP-IL6 mice and age-matched ChAT(BAC)-eGFP of mixed genders at three different age groups corresponding to young (3–4 months, *n = *15), adults (9–12 months, *n = *15), and old (18–24 months, *n = *15). All animals were handled with standard conditions of temperature, humidity, 12-h light/dark cycle, free access to food and water, and without any intended stress stimuli.

All experiments and procedures on mice were approved by the Animal Care and Ethics Committee (ACEC) of Western Sydney University (ACEC Number- A14644, A13230, and A13673). Animals were handled according to the “Guidelines to Promote the Wellbeing of Animals used for Scientific Purposes” as described by the National Health and Medical Research Council of Australia.

### Tissue preparation and immunohistochemistry

For histological analysis, the tissue samples were prepared from all experimental cohorts [methods [37]]. Mice were anaesthetized using isoflurane and transcardially perfused at the end of each time point. The heart was flushed with 60 ml of 0.1 M phosphate buffered saline (PBS) or 0.9% normal saline, followed by 100 ml of 4% paraformaldehyde (PFA) in 0.1 M PBS, using a peristaltic pump. The brains were then removed and post-fixed in 4% PFA, for at least 24 h at 4 °C. Brains were then transferred to 30% sucrose (in 0.1 M PBS solution) for cryoprotection. Once the brains sank to the bottom of the container, they were cut into 50-µm-thick coronal sections using a Leica CM 1950 cryostat. For each brain, 3 series of coronal sections were collected from the olfactory bulb to the posterior medial septum (till the anterior commissure is fully formed). The rest of the brain (from hippocampus to the cerebellum) were cut into 6 series of coronal sections. All the sections were then stored in antifreeze solution (30% ethylene glycol, 30% glycerol in PBS and ultrapure H_2_O) and stored at − 20 °C until use.

The first series of the medial septum sections were used for immunohistochemical assays [37] to visualize cholinergic neurons (except for the ChAT^BAC^-eGFAP brains) and microglia (both ChAT^BAC^ -eGFAP and GFAP-IL6 mice brains). The sections were rinsed three times with 0.1 M PBS, and then incubated for 3 h in 3% normal donkey serum (Abcam, ab138579) (0.1 M PBS; 0.1% Triton X) to block the nonspecific binding sites. Sections were then incubated in primary antibody solution (rabbit anti-Iba1, 019–19741, #CAP4688 from Wako Chemical; 1:500; goat anti-ChAT, ab 144p, #3,429,630 from Merck; 1: 250; 0.1 M PBS; 0.1% Triton X) for 48 h at − 4 °C. Sections were then rinsed with 0.1 M PBS for three times, and incubated in secondary antibody solution (Alexa Fluor 594-conjugated Affinity Pure donkey anti-rabbit IgG for Iba1, 1:200, 711–585-152, #145,043 from Jackson Immuno Research; Alexa Fluor 488-conjugated Affinity Pure donkey anti-goat IgG for ChAT, 1:200, 705–545-003, #129,709 from Jackson Immuno Research) for 2 h at room temperature. Sections were rinsed again and mounted on gelatine-coated slides, and cover slipped with Vectashield mounting medium hard set with DAPI (Vectorlabs, H-1200).

### Stereological counting

The estimated number of Iba-1^+^ microglial cells and ChAT^+^ cholinergic cells in the MS was counted using the Zeiss AxioImager M2 microscope equipped with MBF Biosciences StereoInvestigator [45, 46]. The contour of the MS was first drawn under the 5× objective. It is noteworthy, that the MS volume in context was delineated for stereology measures conferring to the ChAT^+^ cholinergic cell distribution. The sampling grid and the counting frame sizes were 220 × 220 µm and 120 × 120 µm, respectively, for both microglia and cholinergic cells among all four cohorts. The guard zone was 1 µm at the top and the bottom of the sections. Microglia and cholinergic cells were plotted on the screen using different markers as the focus moved from the top to the bottom of the sections using a 63 × oil objective. This led to a Gunderson coefficient error of less than 0.1 in all cases (m = 1). Summary of stereological parameters used to count medial septal Iba-1^+^ microglia and ChAT^+^ cholinergic cell numbers and density, as well as the septal volume are summarized in Additional file 1: Table S1, S2 and S3.

### Three-dimensional reconstruction

Cover-slipped samples stained for both Iba-1^+^ microglial cells and ChAT^+^ cholinergic cells were imaged using a Confocal ZEISS Laser Scanning Microscope (LSM800) with an argon laser and processed using the Zen Blue^®^ software package [method adapted from our previous published work [35]]. The super resolution images were obtained using Airyscan with 1.3 × crop area, and Z-stacks were captured using a 63 × objective and NA1.0 for reflective imaging, at a step size of 0.18–2 µm (unless otherwise specified). Reflective imaging was achieved using the 561 nm wavelength for microglia, and 488 nm wavelength for cholinergic cells (stitched images limited to 4 tiled areas). The 3D reconstruction of microglia, and cholinergic cells were performed using Neurolucida 360^®^ (MBF Bioscience) software. After importing the Z-stacked images of both Iba-1^+^ microglial cells and ChAT^+^ cholinergic cells to Neurolucida 360^®^ software, these cells were manually reconstructed using the user-guided mode, along the required planes, obtaining a reconstructed 3D image of each cell (methods [35, 37]). Extended depth of focus images were obtained by collapsing the Z-stacked 3D images of resolution 1596 × 1596 pixels, to better identify the objects and provide greater accuracy. Six Iba-1^+^ microglial cells per brain (*n = *180 altogether) and five ChAT^+^ cholinergic cells per brain (*n = *150 altogether) were analysed from each cohort of mice at three time points.

### Analysis of reconstructed cells

Morphometric data, of each Iba1^+^ microglial cell and ChAT^+^ cholinergic cell, were extracted with the Neurolucida 360^®^ reconstruction software. Each reconstructed cell was subject to multiple measures using Neurolucida Explorer, the analytical software companion for Neurolucida 360. As described recently by our group, the soma area, µm^2^; soma perimeter, µm; soma circularity (proportional index between cell area and perimeter); processes from soma, n; total length of processes, µm; number of nodes (branch points), n, were measured using Branched Structure analysis [35]. To further confirm the morphological changes displayed with branched structure analysis, the convex-hull analysis was used, that measured the size of arborization field by interpreting a branched structure as a solid object controlling a given amount of physical space. The amount of physical space was then defined in terms of surface area, µm^2^; volume, µm^3^; perimeter, µm. Sholl analysis [47], for each microglial cell and cholinergic cell, was performed to ascertain changes in cell size in relation to the distance from the cell soma [35]. This analysis was performed in nested concentric spheres (radius, r) centred at the cell soma, *r* = 0, with increase in size by a constant change in radius (5 µm steps for Iba-1^+^ microglia; 10 µm steps for ChAT^+^ cholinergic cells) and counting the number of compartments crossing a given radius. Sholl diagrams were averaged, and maximum value was obtained as the maximum number of crossings. This analysis determined the number of intersections, the process length (µm), the surface area of the cells (µm^2^), the process volume (µm^3^), the process diameter (µm), and a number of nodes of the cells for each radius. Summary of the morphological measurements for septal Iba-1^+^ microglia and ChAT^+^ cholinergic cells are summarized in Additional file 1: Tables S4, S5, S6 and S7.

### Golgi dendritic spine analysis

The Golgi–Cox method has been recognized as one the most effective methods for studying the morphology of neuronal dendrites and dendritic spines [48]. The much reliable and efficient (less time consuming) FD Rapid GolgiStain™ Kit [49] was used in this study, to examine changes in hippocampal CA1 pyramidal spine density. Fresh brain tissue was collected (*n = *2, per animal cohort) and cryosectioned at 100 µm, for Golgi impregnation, and followed exactly the manufacturer's specifications (FD Rapid GolgiStain Kit). Golgi-stained hippocampal tissue was observed using a Zeiss M2 bright-field microscope with StereoInvestigator (MBF Bioscience, Version10) to locate hippocampal CA1 pyramidal cells based on their unique morphology. Pyramidal dendritic spines (*n = *20, dendritic branches per cohort) were imaged at 60× oil objective, with a desired height of 20 µm and 1 μm step distance. The images were then processed using Neurolucida360 (MBF Bioscience) to generate 3D reconstructions of the desired dendritic branch with spines, and in-built Neurolucida Explorer (MBF Bioscience) was used to provide spine density analysis of these reconstructions. Spine density measurements are summarized in Additional file 1: Table S8.

### Qualitative representation of degenerating fibres in the medial septum

This assessment was conducted using one series from the 3 series of 50 µm coronal sectioned mouse brains (from post-fixed brains described in section; Tissue Preparation and Immunohistochemistry), from each of the 6 cohorts. The MS was silver-stained by FD NeuroSilver™ Kit II (from FD Neurotechnologies, Inc., Columbia-US) following the manufacturer's protocol, to detect the degenerating fibres in this brain region, as previously described for other brain regions [50, 51]. Images were captured with the Carl Zeiss-upright bright-field microscope-Axio Imager M2 (Carl Zeiss AG, Germany), at 20× magnification. When necessary, contrast and brightness was adjusted using Adobe Photoshop 2023.

### Slice preparations

Animals were deeply anesthetized by inhalation of isoflurane (5%) followed by injection with ketamine/xylazine cocktail (100 mg/kg and 200 mg/kg, respectively). Following anesthesia, mice were transcardially perfused with ice-cold artificial CSF (aCSF) containing (in mM): 125 NaCl, 2.5 KCl, 1 MgCl_2_, 1.25 NaH_2_PO_4_, 2 CaCl_2_, 25 NaHCO_3_, 12.5 glucose and saturated with carbogen (95% O_2_ − 5% CO_2_ mixture; pH 7.4), until the outflow solution was clear. Following perfusion, mice were decapitated, the brains were quickly removed and placed into ice-cold aCSF (as above). Coronal brain slices (300 μm thick) were cut with a vibrating microtome (Leica VT1200S) and transferred to the Braincubator™ (PaYo Scientific, Sydney; http://braincubator.com.au), as reported previously [52]. The Braincubator™ is an incubation system that closely monitors and controls pH, carbogen flow, and temperature, as well as irradiating bacteria through a separate UV chamber [53–55]. Slices were initially incubated for 12 min at 35 °C, after which they were allowed to cool to 15–16◦C and kept in the Braincubator™ for at least 30 min before measurements started.

### Electrophysiological recording and stimulation

The recording chamber was mounted on an Olympus BX-51 microscope equipped with IR/DIC optics. Following incubation in the Braincubator™, slices were mounted in the recording chamber for a minimum of 5 min, to allow them to be warmed to 36 °C and were constantly perfused at a rate of 2 ml/min with carbogenated aCSF, as reported previously [56]. Whole-cell intracellular recordings from ChAT-eGFP positive neurons in the medial septum were obtained with patch pipettes (5–7 MΩ) containing (in mM): 126 K-gluconate, 4 KCl, 10 HEPES, 0.3 EGTA, 10 phosphocreatine disodium salt hydrate, 0.3 Na-GTP, 4 Mg-ATP, and titrated with KOH to pH 7.2 (∼ 285 mOsm). Voltages were recorded in current clamp mode using a multiclamp 700B dual patch-clamp amplifier (Molecular Devices), digitally sampled at 30–50 kHz, filtered at 10 kHz, and analysed off-line using pClamp 10 software [57, 58]. Cells were considered stable and suitable for analysis if the access resistance and resting membrane potential did not change by more than 20% from their initial value during recording.

### Suprathreshold sinusoidal stimulus protocol

In order to evaluate alterations in suprathreshold oscillation frequencies under different conditions, we measured the sinusoidal–frequency curves as reported previously [59]. In short, 10-s stimulating protocols of sinusoidal current (chirp stimulation), in which there was a linear increase in the frequency from 0.1 to 100 Hz, were delivered at 30, 60, 125 and 250 pA, using the pClamp 10 software suite (Molecular Devices, Sunnyvale, CA, USA) and injected into the neuronal soma through the recording electrode.

### Data analysis

Analysis of variance (ANOVA) was performed for all comparisons between cohorts. Two-way ANOVA was used to compare outputs from the optical fractionator, branched structural analyses, and convex-hull analysis, followed by Tukey’s post hoc tests to detect ‘age’ and ‘genotype’ effects. Significance was determined by *p* < 0.05, and where Tukey’s multiple comparison test was used to determine the post hoc effect for differences between means (MD), significance of ‘genotype’ was represented by asterisks (*p < 0.05, **p < 0.01, ***p < 0.001, ****p < 0.0001); and ‘age’ was represented by hash symbols (^#^*p* < 0.05, ^##^*p* < 0.01, ^###^*p* < 0.001, ^####^*p* < 0.0001). Prism GraphPad (Version 8) and Microsoft Excel were used to conduct these statistical analyses. Sholl analysis data were compared using one-way ANOVA for area under the curve (AUC) data of cumulative line graphs for each measurement, followed by Tukey’s post hoc tests to detect ‘age’ and ‘genotype’ effects. All morphological data are presented as mean ± SEM.

All electrophysiological recordings were analysed with two-way ANOVA test followed by Tukey’s post hoc tests to detect ‘age’ and ‘genotype’ effects. Data are shown as box plot, the box upper and lower limits are the 25th and 75th quartiles, respectively. The whiskers depict the lowest and highest data points, the horizontal line through the box is the median and the + sign represents the mean. Post hoc effect of ‘genotype’ indicated by asterisks, (*p < 0.05, **p < 0.01, ***p < 0.001, ****p < 0.0001); post hoc effect of ‘age’ indicated by hash symbols (^#^*p* < 0.05, ^##^*p* < 0.01, ^###^*p* < 0.001, ^####^*p* < 0.0001).

## Results

### Aging and chronic neuroinflammation are associated with increased reactive microglia in the medial septum and reduced cholinergic cell number

**Fig. 1:**
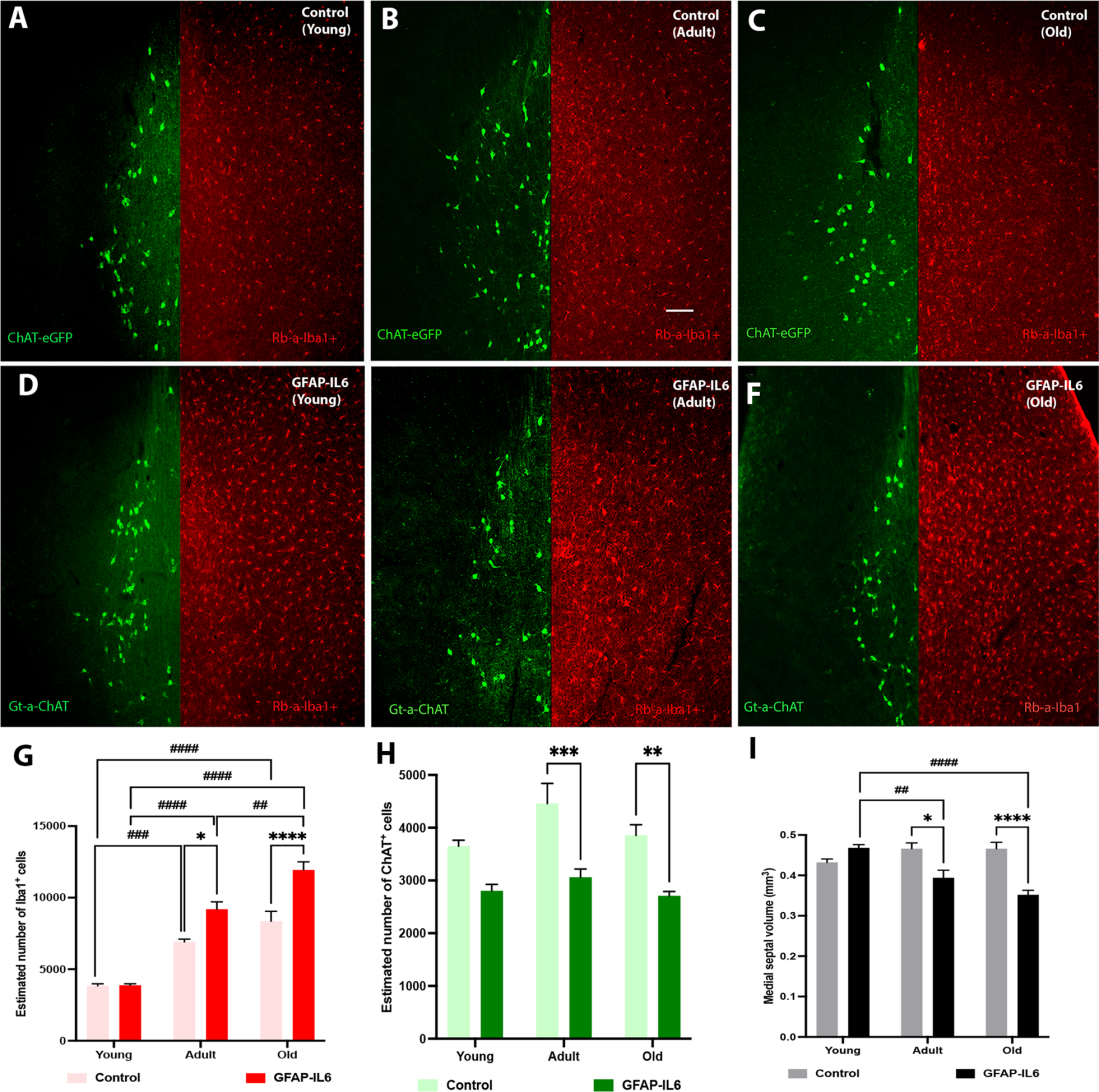
The number of Iba-1^+^ microglia and ChAT^+^ cholinergic cells in the medial septum (MS) changes with age and chronic neuroinflammation. **A**–**F** Representative fluorescence microscopy images comparing changes of the Iba1^+^ microglia and ChAT^+^ cholinergic cell population in the medial septum (MS) during aging and neuroinflammation. The images displaying the changes in ChAT^+^ (green) and Iba1^+^ (red) cells in the MS for, young, adult, and old mice from control cohorts (**A**–**C**) and GFAP-IL6 cohorts (**D**–**F**). Images taken under the 10 × objective using green and red channels to detect ChAT^+^ cholinergic cells and Iba1^+^ microglia cells, respectively (scale bar 100 µm). **G**–**I** Grouped bar graphs representing the stereological estimation changes and septal volume, with aging and neuroinflammation; **G** septal Iba-1^+^ microglia number, **H** septal ChAT^+^ cell number, and **I** septal volume, of young (*n = *5), adult (*n = *5), and old (*n = *5) mice from control and GFAP-IL6 cohorts. Data presented as mean ± SEM; two-way ANOVA, with Tukey’s post hoc test. Post hoc effect of ‘genotype’ indicated by asterisks, (*p < 0.05, **p < 0.01, ***p < 0.001, ****p < 0.0001); post hoc effect of ‘age’ indicated by hash symbols (^#^*p* < 0.05, ^##^*p* < 0.01, ^###^*p* < 0.001, ^####^*p* < 0.0001)

To investigate the effect of normal versus pathological aging, we performed immunohistochemical analysis on the Iba-1^+^ microglia and ChAT^+^ cholinergic cell population in the MS, at three different age groups corresponding to young, adult and old, in both genotypes. The qualitative differences observed from fluorescence microscopy imaging (Fig. 1A–F) were confirmed using appropriate statistical analysis.

A significant main effect of ‘age’ and ‘genotype’, as well as ‘age’ x ‘genotype’ interaction was observed on the level of microglia activation in the medial septum [‘age’ F (2, 24) = 107.8, *P* < 0.0001; ‘genotype’ F (1, 24) = 30.54, *P* < 0.0001; ‘age’ x ‘genotype’ F (2, 24) = 8.378, *P* = 0.0017]. Post hoc test confirmed a significant increase in estimated septal Iba-1^+^ microglia number in the control cohort for adult (57.20%; MD = 3073 ± 616.8, ^###^*p* = 0.0006) and old (74.19%; MD = 4524 ± 616.8, ^####^*p* < 0.0001) compared to young control cohort (Fig. 1G; Additional file 1: Table S3). A much-exaggerated significant increase was observed in adult (81.20%; MD = 5307 ± 616.8, ^####^*p* < 0.0001) and old (101.82%; MD = 8053 ± 616.8, ^####^*p* < 0.0001) GFAP-IL6 cohorts compared to young GFAP-IL6 cohort. We also observed a significant increase in Iba-1^+^ microglia in old GFAP-IL6 cohort compared to adult GFAP-IL6 cohort (26%; MD = 2746 ± 616.8, ^##^*p* = 0.0021). Importantly significant differences were observed between adult (28.34%; MD = 2281 ± 616.8, ^*^p = 0.0127) and old (35.24%; MD = − 3576 ± 616.8, ^****^p < 0.0001) GFAP-IL6 cohorts and their age-matched controls, but no significant difference for young cohorts between the two genotypes.

We observed a significant main effect of both ‘age’ and ‘genotype’ on the septal ChAT^+^ cholinergic cell number, with no significant ‘age’ x ‘genotype’ interaction [‘age’ F (2, 24) = 5.187, *P* = 0.0134; ‘genotype’ F (1, 24) = 18.88, *P* = 0.0002]. Post hoc test confirmed a significant decrease in ChAT^+^ cholinergic cell number in adult (37.20%; MD = − 1399 ± 285.7, ^***^p = 0.0007) and old (34.96%; MD = − 1148 ± 285.7, ^**^p = 0.0059) GFAP-IL6 cohorts compared to their age-matched control cohorts (Fig. 1H; Additional file 1: Table S3).

A significant main effect of ‘age’ and ‘genotype’, as well as ‘age’ × ‘genotype’ interaction was observed for the medial septal volume across the cohorts [‘age’ F (2, 24) = 4.670, *P* = 0.0194; ‘genotype’ F (1, 24) = 20.83, *P* = 0.0001; ‘age’ × ‘genotype’ F (2, 24) = 16.63, *P* < 0.0001]. Post hoc test confirmed a significant decrease in septal volume in adult (18.60%; MD = − 0.07400 ± 0.01897, ^##^*p* = 0.0079) and old (29.27%; MD = − 0.1160 ± 0.01897, ^####^*p* < 0.0001) GFAP-IL6 cohorts compared to young GFAP-IL6 cohort (Fig. 1I; Additional file 1: Table S3). We did not observe a significant septal volume loss across normal aging cohorts. However, a significant decrease in septal volume was seen in adult (18.60%; MD = − 0.07200 ± 0.01897, ^*^p = 0.0101) and old (29.27%; MD = − 0.1140 ± 0.01897, ^****^p < 0.0001) GFAP-IL6 cohorts compared to their age-matched control cohorts.

### Aging and chronic neuroinflammation are associated with morphological changes in microglia in the medial septum

**Fig. 2:**
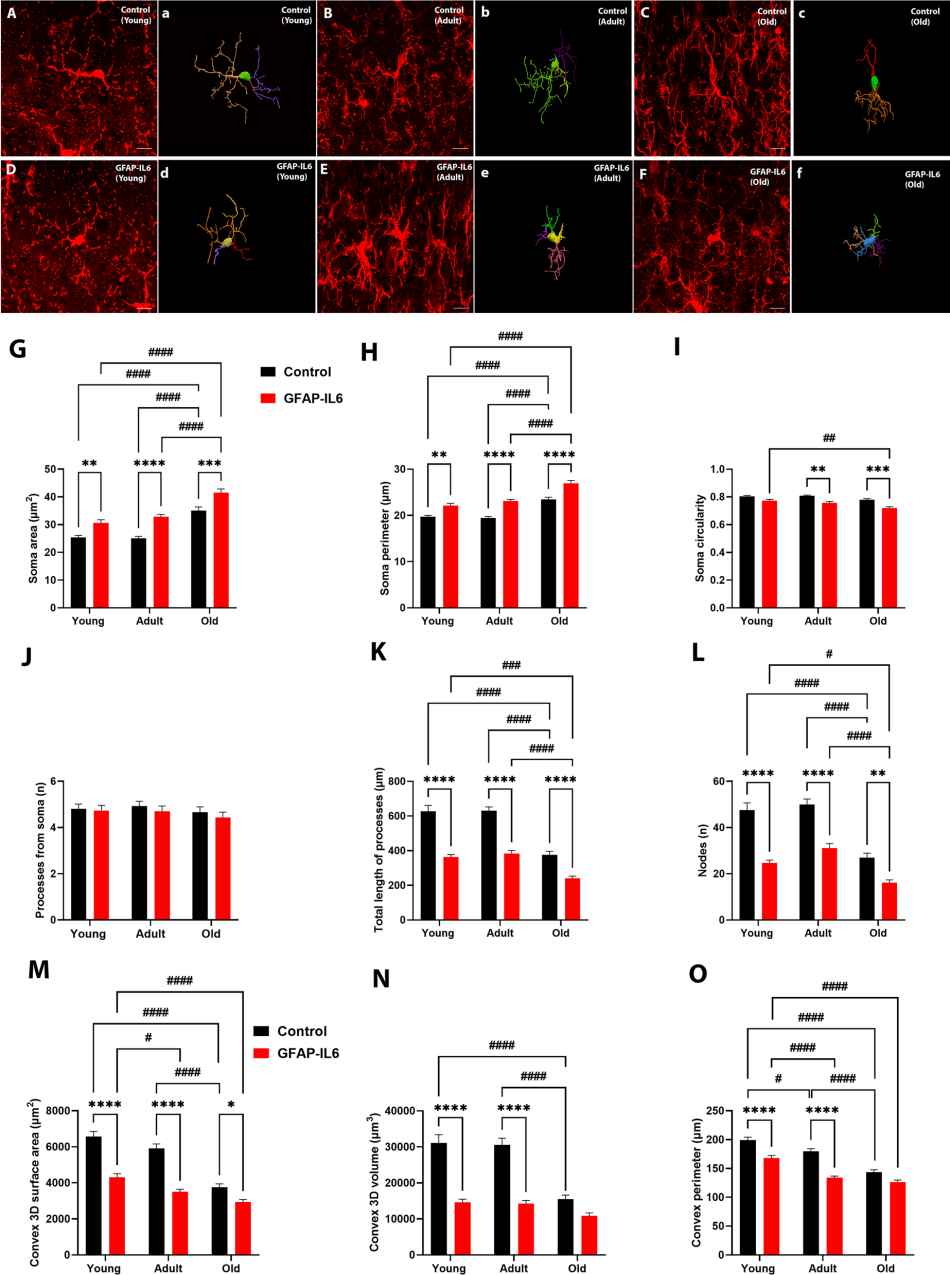
Morphology of septal Iba-1-positive microglia during aging and the impact of chronic neuroinflammation. **A**–**E** Representative confocal images of reconstructed medial septum (MS) microglia from control and GFAP-IL6 cohorts. The extended depth of focus images (A-E) obtained from collapsing 3D confocal microscopy images of Iba-1 + microglia obtained under 63 × oil immersion objective, along with the corresponding manually reconstructed images (a–e) in Neurolucida 360, of young, adult and old mice from control and GFAP-IL6 cohorts (scale bar 10 μm). **G**–**L** Grouped bar graphs representing the effects of aging and neuroinflammation on Iba-1^+^ microglial cell morphology in the medial septum (MS), measured by branched structure analysis. Quantitative analysis of the morphological changes in Iba-1^+^ microglia, including **G** soma area (µm^2^), H soma perimeter (µm), I soma circularity, J number of processes from soma (n), K total length of processes (µm) and L number of nodes (n), of young (*n = *30), adult (*n = *30) and old (*n = *30) mice from control (black) and GFAP-IL6 (red) cohorts. Data presented as mean ± SEM and analysed with two-way ANOVA followed by Tukey’s post hoc tests. Post hoc effects of ‘genotype’ are represented in asterisks (*p < 0.05, **p < 0.01, ***p < 0.001, ****p < 0.0001); post hoc effects of ‘age’ are represented by hash symbols (^#^*p* < 0.05, ^##^*p* < 0.01, ^###^*p* < 0.001, ^####^*p* < 0.0001). **M–O** Grouped bar graphs representing the effects of aging and neuroinflammation on Iba-1^+^ microglial cell spatial distribution in the medial septum (MS), measured by convex-hull analysis. Quantitative analysis of spatial distribution changes in Iba-1^+^ microglia, including **M** convex 3D surface area (µm^2^), **N** convex 3D volume (µm^3^) and **O** convex perimeter (µm) of young (*n = *30), adult (*n = *30) and old (*n = *30) mice from control (black) and GFAP-IL6 (red) cohorts. Data presented as mean ± SEM and analysed with two-way ANOVA followed by Tukey’s post hoc tests. Post hoc effects of ‘genotype’ are represented in asterisks (*p < 0.05, **p < 0.01, ***p < 0.001, ****p < 0.0001); post hoc effects of ‘age’ are represented by hash symbols (^#^*p* < 0.05, ^##^*p* < 0.01, ^###^*p* < 0.001, ^####^*p* < 0.0001)

Following up on the significant stereological differences observed for septal Iba1^+^ microglia and ChAT^+^ cholinergic cell populations, morphological analysis was conducted. To further understand the impact of aging and neuroinflammation on the morphological features of the septal Iba-1^+^ microglia, single cell branched structural analysis of young, adult, and old mice from control and GFAP-IL6 cohorts was performed (Fig. 2A–F).

A significant main effect of ‘age’ and ‘genotype’ was observed in the measurements of soma area (µm^2^) [‘age’ F (2, 58) = 51.70, *P* < 0.0001; ‘genotype’ F (1, 29) = 60.37, *P* < 0.0001], soma perimeter (µm) [‘age’ F (2, 58) = 59.09, *P* < 0.0001; ‘genotype’ F (1, 29) = 88.09, *P* < 0.0001], and soma circularity [‘age’ F (2, 58) = 13.35, *P* < 0.0001; ‘genotype’ F (1, 29) = 54.69, *P* < 0.0001]. However, we did not observe ‘age’ × ‘genotype’ interaction for these measurements. Post hoc test confirmed significant increase in soma area and perimeter, respectively (Fig. 2G, H; Additional file 1: Supplemetary Table S4) in young (MD 5.248 ± 1.368, ^**^p = 0.0040; MD 2.397 ± 0.6270, ^**^p = 0.0042), 12- (MD 7.757 ± 1.368, ^****^p < 0.0001; MD 3.664 ± 0.6270, ^****^p < 0.0001), and old (MD 6.457 ± 1.368, ^***^p = 0.0002; MD 3.500 ± 0.6270, ^****^p < 0.0001) GFAP-IL6 cohorts compared to age-matched control cohorts. There was also a significant increase in soma area and perimeter, respectively, for old control compared to young control cohort (MD 9.677 ± 1.368, ^####^*p* < 0.0001; MD -3.763 ± 0.6270, ^####^*p* < 0.0001), as well as between old control compared to adult control cohort (MD 9.979 ± 1.368, ^####^*p* < 0.0001; MD -3.983 ± 0.6270, ^####^*p* < 0.0001). This significant increase in soma area and perimeter was higher between young and old GFAP-IL6 cohorts (MD 10.89 ± 1.368, ^####^*p* < 0.0001; MD 4.866 ± 0.6270, ^####^*p* < 0.0001), as well as for adult and old GFAP-IL6 cohorts (MD -8.679 ± 1.368, ^####^*p* < 0.0001; MD; 3.819 ± 0.6270, ^####^*p* < 0.0001). Furthermore, we observed a significant reduction in soma circularity (Fig. 2I) in adult (MD − 0.05100 ± 0.01376, ^**^p = 0.0060) and old (MD -0.05967 ± 0.01376, ^***^p = 0.0008) GFAP-IL6 cohorts compared to their age-matched control cohorts, as well as in old GFAP-IL cohort compared to young GFAP-IL6 cohort (MD -0.05533 ± 0.01376, ^##^*p* = 0.0022). There was no significant effect of ‘age’ or ‘genotype’ on the number of processes form Iba-1^+^ septal microglia (Fig. 2J). However, we found a significant main effect of ‘age’, ‘genotype’, and ‘age’ x ‘genotype’ interaction, respectively, for total length of processes from soma (µm) [F (2, 58) = 73.91, *P* < 0.0001; F (1, 29) = 99.10, *P* < 0.0001; F (2, 58) = 6.581, *P* = 0.0027] and nodes (n) [F (2, 58) = 67.69, *P* < 0.0001; F (1, 29) = 68.89, *P* < 0.0001; F (2, 58) = 4.535, *P* = 0.0148]. Post hoc test confirmed significant decrease in total length of processes and nodes, respectively (Fig. 4K, L; Additional file 1: Supplementary Table S4) in young (MD − 263.9 ± 27.17, ^****^p < 0.0001; MD − 22.73 ± 2.842, ^****^p < 0.0001), adult (MD− 247.0 ± 27.17, ^****^p < 0.0001; MD − 18.87 ± 2.842, ^****^p < 0.0001), and old (MD − 135.6 ± 27.17, ^****^p < 0.0002; MD − 10.87 ± 2.842, ^**^p = 0.0042) GFAP-IL6 cohorts compared to age-matched control cohorts. Moreover, there was a significant decrease in in total length of processes and nodes, respectively, within the aging cohorts for both control and GFAP-IL6 cohorts between: young and old control cohorts (MD − 250.9 ± 27.17, ^####^*p* < 0.0001; MD − 20.53 ± 2.842, ^####^*p* < 0.0001); adult and old control cohorts (MD -254.4 ± 27.17, ^####^*p* < 0.0001; MD − 23.03 ± 2.842, ^####^*p* < 0.0001); young and old GFAP-IL6 cohorts (MD − 122.6 ± 27.17, ^###^*p* = 0.0004; MD − 8.667 ± 2.842, ^#^*p* = 0.0385); adult and old GFAP-IL6 cohorts (MD − 143.0 ± 27.17, ^####^*p* < 0.0001; MD − 15.03 ± 2.842, ^####^*p* < 0.0001).

To further confirm the morphological changes displayed with branched structure analysis, the convex-hull analysis was performed. There was a significant main effect of ‘age’ and ‘genotype’ as well as ‘age’ x ‘genotype’ interaction, respectively, for the measurements of convex 3D surface area (µm^2^) [F (2, 58) = 56.25, *P* < 0.0001; F (1, 29) = 87.82, *P* < 0.0001; F (2, 58) = 11.72, *P* < 0.0001], volume (µm^3^) [F (2, 58) = 32.78, *P* < 0.0001; F (1, 29) = 83.08, *P* < 0.0001; F (2, 58) = 14.36, *P* < 0.0001], and perimeter (µm) [F (2, 58) = 67.15, *P* < 0.0001; F (1, 29) = 69.29. *P* < 0.0001; F (2, 58) = 5.492, *P* = 0.0065] (Fig. 2; Additional file 1: Supplementary Table S4). Post hoc test confirmed significant reduction in microglia arborization field in terms of surface area (Fig. 2M) for young (MD -2269 ± 255.0, ^****^p < 0.0001), adult (MD -2399 ± 255.0, ^****^p < 0.0001), and old (MD − 826.4 ± 255.0, ^*^p = 0.0231) GFAP-IL6 cohorts compared to their age-matched control cohorts. There was also a significant reduction in surface area within the aging cohorts between; young and old control cohorts (MD − 2809 ± 255.0, ^####^*p* < 0.0001); adult and old control mice (MD − 2146 ± 255.0, ^####^*p* < 0.0001); young and adult GFAP-IL6 cohorts (MD − 791.9 ± 255.0, ^#^*p* = 0.0332); young and old GFAP-IL6 cohorts (MD − 1366 ± 255.0, ^####^*p* < 0.0001). In terms of volume (Fig. 2N), we found a significant reduction in young (MD − 16,530 ± 1781, ^****^p < 0.0001), and adult (MD − 16,219 ± 1781, ^****^p < 0.0001) GFAP-IL6 cohorts compared to age-matched control cohorts. A significant reduction in volume within the aging cohorts were visible between; young and old control cohorts (MD − 15,592 ± 1781, ^####^*p* < 0.0001); adult and old control cohorts (MD − 14,984 ± 1781, ^####^*p* < 0.0001). Furthermore, in terms of perimeter (Fig. 2O), a significant reduction was found in young (MD -31.41 ± 6.003, ^****^p < 0.0001), and adult (MD − 45.44 ± 6.003, ^****^p < 0.0001) GFAP-IL6 cohorts compared to age-matched control cohort. There was also a significant reduction in perimeter (µm) within the aging cohorts between; young and adult control cohorts (MD − 19.57 ± 6.003, ^#^*p* = 0.0219); young and old control cohorts (MD − 55.55 ± 6.003, ^####^*p* < 0.0001); adult and old control cohorts (MD -35.98 ± 6.003, ^####^*p* < 0.0001); young and adult GFAP-IL6 cohorts (MD -33.60 ± 6.003, ^####^*p* < 0.0001); young and old GFAP-IL6 cohorts (MD − 41.45 ± 6.003, ^####^*p* < 0.0001).

**Fig. 3:**
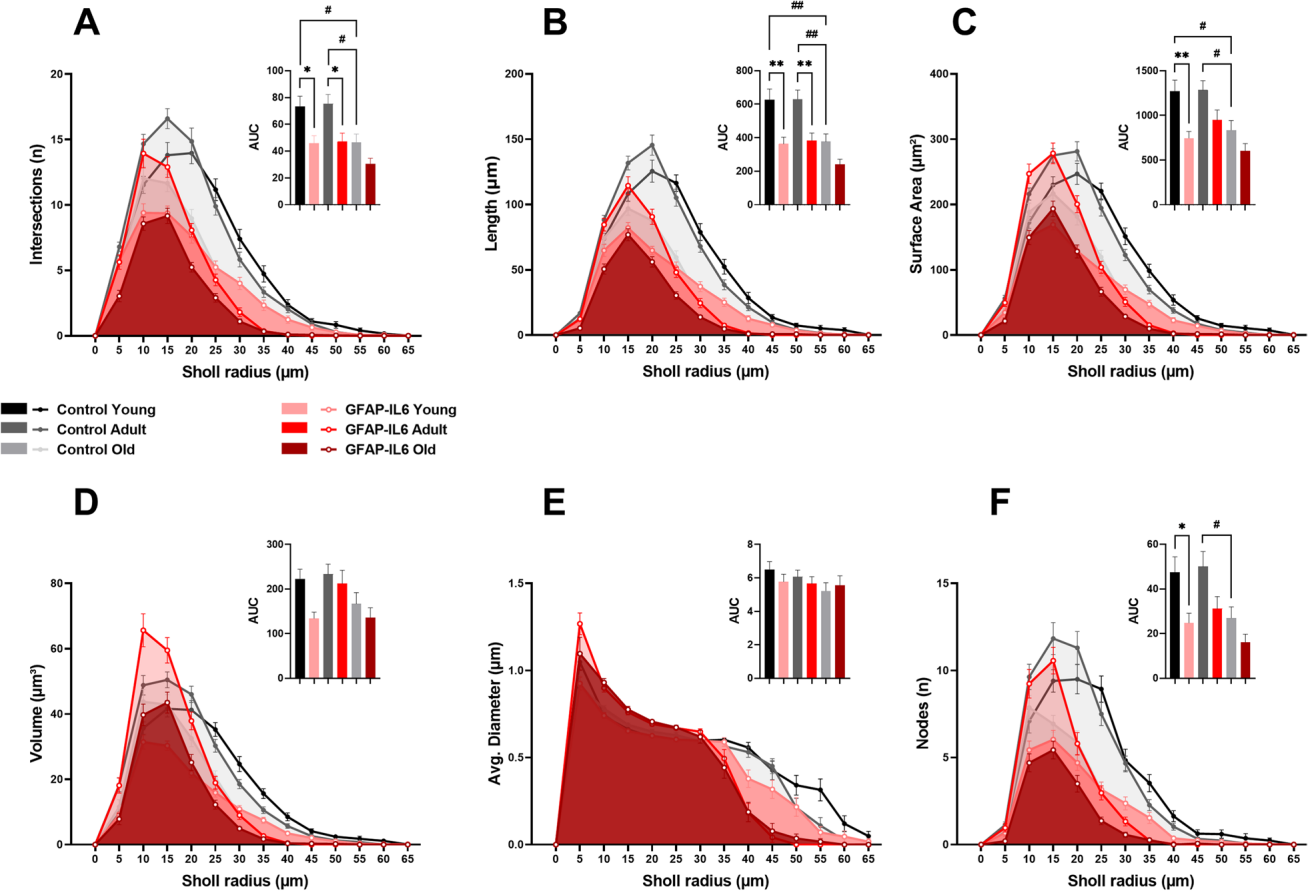
Cumulative line graphs and corresponding area under the curve (AUC) bar graphs, representing the effects of aging and neuroinflammation on Iba-1^+^ microglial cell arborization complexity in the medial septum (MS), measured by Sholl analysis. Quantitative analysis of Iba-1^+^ microglia arborization complexity, including **A** intersections (n), **B** length (µm), **C** surface area (µm^2^), **D** volume (µm^3^), **E** average diameter (µm), and **F** nodes (n) of young (*n = *30), adult (*n = *30) and old (*n = *30) mice from control and GFAP-IL6 cohorts. Data presented as mean ± SEM for AUC graphs and analysed with one-way ANOVA followed by Tukey’s post hoc tests. Post hoc effects of ‘genotype’ are represented in asterisks (*p < 0.05, **p < 0.01, ***p < 0.001, ****p < 0.0001); post hoc effects of ‘age’ are represented by hash symbols (^#^*p* < 0.05, ^##^*p* < 0.01, ^###^*p* < 0.001, ^####^*p* < 0.0001)

Finally, to further investigate the effects of aging and neuroinflammation on the complexity of microglia arborization, a segmental (Sholl) analysis was performed to examine the changes as a function of radial distance from the microglia cell body. We observed less complex microglial arborization in GFAP-IL6 mice with aging, mostly confined to 0–30 µm radius, compared to age-matched control mice which showed interactions beyond the 30 µm radius (Fig. 3). After analysing the area under the curve (AUC) for each cumulative line graph (Fig. 3A–F; Additional file 1: Supplementary Table S5), overall significant mean peak differences were observed between GFAP-IL6 mice and their age-matched controls for the measurements of intersections (n) [F (5, 2424) = 7.781, *P* < 0.0001], length (µm) [F (5, 2424) = 10.80, *P* < 0.0001], surface area (µm^2^) [F (5, 2424) = 7.311, *P* < 0.0001], volume (µm^3^) [F (5, 2424) = 3.599, *P* = 0.0030], and nodes (n) [F (5, 2424) = 5.955, *P* < 0.0001]. No significant differences were observed among the cohorts for the average diameter (µm). Post hoc comparisons displayed a significant reduction in AUC for intersections (Fig. 3A) in young (MD -27.30 ± 8.888, ^*^p = 0.0262) and adult (MD − 28.24 ± 8.888, ^*^p = 0.0188) GFAP-IL6 cohorts when compared to their age-matched control cohorts. A significant reduction was also observed among the aging cohorts between young and old control cohorts (MD − 26.63 ± 8.888, ^#^*p* = 0.0329), and adult and old control cohorts (MD − 28.67 ± 8.888, ^#^*p* = 0.0161). Measurements of AUC for length (Fig. 3B) displayed a significant reduction in young (MD − 264.0 ± 7.81, ^**^p = 0.0014) and adult (MD − 247.0 ± 67.81 ± 8.888, ^**^p = 0.0037) GFAP-IL6 cohorts when compared to their age-matched control cohorts. A significant length reduction was also observed among the aging cohorts between young and old control cohorts (MD − 250.8 ± 67.81, ^##^*p* = 0.0030), and adult and old control cohorts (MD -254.4 ± 67.81, ^##^*p* = 0.0025). We also observed a significant reduction in AUC for surface area (Fig. 3C) in young GFAP-IL6 cohort compared to their age-matched control cohort (MD − 524.0 ± 145.8, ^**^p = 0.0045). Moreover, a significant reduction in surface area was observed among the aging cohorts between young and old control cohorts (MD − 434.0 ± 145.8, ^#^*p* = 0.0348), and adult and old control cohorts (MD -448.0 ± 145.8, ^#^*p* = 0.0261). Furthermore, there was a significant reduction in the number of nodes (Fig. 3F) in the young GFAP-IL6 cohort compared to young control cohort (MD − 22.73 ± 7.741, ^*^p = 0.0393), as well as significant reduction between adult and old control cohorts (MD − 23.03 ± 7.741, ^#^*p* = 0.0350).

### Aging and chronic neuroinflammation are associated with morphological changes in cholinergic cells in the medial septum

**Fig. 4:**
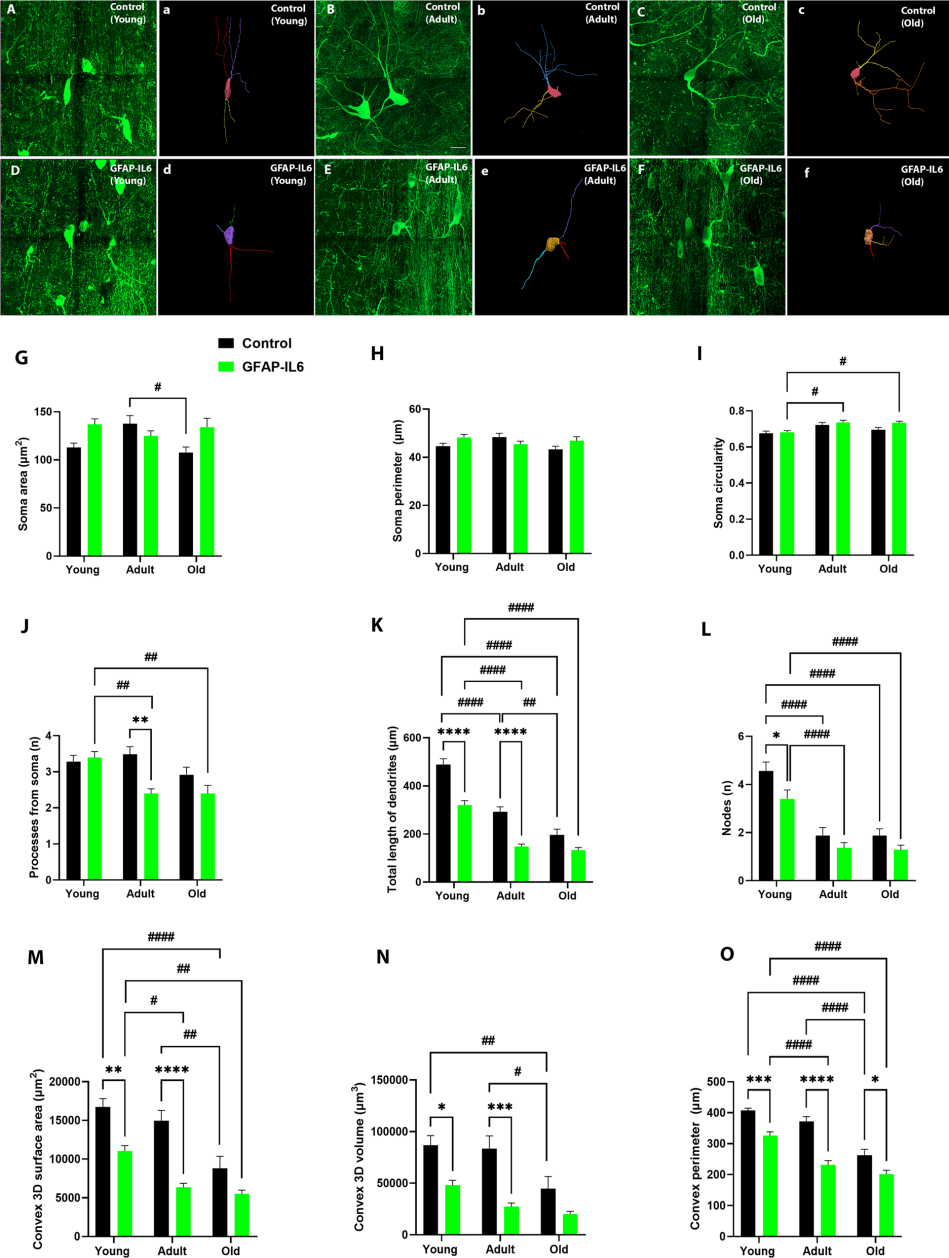
Morphology of septal ChAT-positive neurons during aging and the impact of chronic neuroinflammation. **A**–**F** Representative confocal images of reconstructed medial septum (MS) cholinergic cells from control and GFAP-IL6 cohorts. The extended depth of focus images (**A**–**E**) obtained from collapsing 3D tile scanned confocal microscopy images of ChAT + cholinergic cells obtained under 63 × oil immersion objective, along with the corresponding manually reconstructed images (a-e) in Neurolucida 360, of young, adult and old mice from control and GFAP-IL6 cohorts (scale bar 20 μm). **G**–**L** Grouped bar graphs representing the effects of aging and neuroinflammation on ChAT^+^ cholinergic cell morphology in the medial septum (MS), measured by branched structure analysis. Quantitative analysis of the morphological changes in ChAT^+^ cholinergic cells, including **G** soma area (µm^2^), **H** soma perimeter (µm), **I** soma circularity, **J** number of processes from soma (n), **K** total length of processes (µm) and **L** number of nodes (n), of young (*n = *25), adult (*n = *25) and old (*n = *25) mice from control (black) and GFAP-IL6 (green) cohorts. Data presented as mean ± SEM and analysed with two-way ANOVA followed by Tukey’s post hoc tests. Post hoc effects of ‘genotype’ are represented in asterisks (*p < 0.05, **p < 0.01, ***p < 0.001, ****p < 0.0001); post hoc effects of ‘age’ are represented by hash symbols (^#^*p* < 0.05, ^##^*p* < 0.01, ^###^*p* < 0.001, ^####^*p* < 0.0001). **M–O** Grouped bar graphs representing the effects of aging and neuroinflammation on ChAT^+^ cholinergic cell spatial distribution in the medial septum (MS), measured by convex-hull analysis. Quantitative analysis of spatial distribution changes in ChAT^+^ cholinergic cells, including **M** convex 3D surface area (µm^2^), **N** convex 3D volume (µm^3^) and **O** convex perimeter (µm) of young (*n = *25), adult (*n = *25) and old (*n = *25) mice from control (black) and GFAP-IL6 (green) cohorts. Data presented as mean ± SEM and analysed with two-way ANOVA followed by Tukey’s post hoc tests. Post hoc effects of ‘genotype’ are represented in asterisks (*p < 0.05, **p < 0.01, ***p < 0.001, ****p < 0.0001); post hoc effects of ‘age’ are represented by hash symbols (^#^*p* < 0.05, ^##^*p* < 0.01, ^###^*p* < 0.001, ^####^*p* < 0.0001)

To further interrogate the impact of aging and chronic neuroinflammation on the morphological differences of the septal cholinergic neurons (Fig. 4A–F), we performed quantitative morphological measurements, including branch structure, convex-hull, and Sholl analysis.

We observed a significant main effect of ‘age’, ‘genotype’, and ‘age’ x ‘genotype’ interaction, respectively, in the ChAT^+^ cell measurements of processes from soma (n) [‘age’ F (2, 48) = 7.129, *P* = 0.0019; ‘genotype’ F (1, 24) = 10.75, *P* = 0.0032; ‘age’ x ‘genotype’ F (2, 48) = 5.152, *P* = 0.0094], and total length of dendrites (µm) [‘age’ F (2, 48) = 72.87, *P* < 0.0001; ‘genotype’ F (1, 24) = 70.24, *P* < 0.0001; ‘age’ x ‘genotype’ F (2, 48) = 4.981, *P* = 0.0108]. A significant main effect of only ‘genotype’ [F (1, 24) = 8.076, *P* = 0.0090] and ‘age’ x ‘genotype’ interaction [F (2, 48) = 5.570, *P* = 0.0067] was observed for soma area (µm^2^). Cholinergic cell soma perimeter was not significantly affected by ‘age’ or ‘genotype’. However, we observed a significant main effect of ‘age’ and ‘genotype’ on soma circularity [‘age’ F (2, 48) = 11.15, *P* = 0.0001; ‘genotype’ F (1, 24) = 4.825, *P* = 0.0379], and nodes (n) [‘age’ F (2, 48) = 34.83, *P* < 0.0001; ‘genotype’ F (1, 24) = 8.101, *P* = 0.0089], with no significant ‘age’ x ‘genotype’ interaction. Post hoc test confirmed significant decrease in ChAT^+^ cholinergic cell soma area (Fig. 4G) only in old control compared to adult control cohort (MD − 29.96 ± 9.295, ^#^*p* = 0.0261). Soma circularity (Fig. 4I) displayed a significant increase in adult (MD 0.05559 ± 0.01677, ^#^*p* = 0.0204) and old (MD 0.05305 ± 0.01677, ^#^*p* = 0.0304) compared to young GFAP-IL6 cohort. A significant decrease was observed for the number of processes from soma (Fig. 4J), between adult control and adult GFAP-IL6 cohorts (MD − 1.080 ± 0.2645, ^**^p = 0.0022); young and adult GFAP-Il6 cohorts (MD − 1.000 ± 0.2645, ^##^*p* = 0.0055); young and old GFAP-IL6 cohorts (MD − 1.000 ± 0.2645, ^##^*p* = 0.0055). Importantly, we observed a significant decrease in total length of dendrites (Fig. 4K) in young (MD -167.7 ± 24.80, ^****^p < 0.0001) and adult (MD − 144.4 ± 24.80, ^****^p < 0.0001) GFAP-IL6 cohorts, respectively, compared to their age-matched control cohorts. There was also a significant decrease in total length of dendrites within the aging cohorts for both control and GFAP-IL6 cohorts between; young and adult control cohorts (MD − 195.8 ± 24.80, ^####^*p* < 0.0001); young and old control cohorts (MD − 292.3 ± 24.80, ^####^*p* < 0.0001); adult and old control cohorts (MD − 96.51 ± 24.80, ^##^*p* = 0.0039); young and adult GFAP-IL6 cohorts (MD − 172.4 ± 24.80, ^####^*p* < 0.0001); young and old GFAP-IL6 cohorts (MD -186.9 ± 24.80, ^####^*p* < 0.0001). Complementary, there was a significant decrease in nodes (Fig. 4L) in young GFAP-IL6 cohort compared to their age-matched control cohort (MD − 1.160 ± 0.3711, ^*^p = 0.0336). Moreover, we observed a significant decrease in nodes within the aging cohorts for both control and GFAP-IL6 mice between young and adult control cohorts (MD − 2.680 ± 0.3711, ^####^*p* < 0.0001); young and old control cohorts (MD − 2.680 ± 0.3711, ^####^*p* < 0.0001); young and adult GFAP-IL6 cohorts (MD − 2.040 ± 0.3711, ^####^*p* < 0.0001); young and old GFAP-IL6 cohorts (MD − 2.120 ± 0.3711, ^####^*p* < 0.0001) (see Additional file 1: Supplementary Table S6).

The convex-hull analysis further confirmed changes to the septal ChAT^+^ cholinergic cell dendritic arbor field size (Fig. 4M–O). We observed a significant main effect of ‘age’ and ‘genotype’ for the measurements of convex 3D surface area (µm^2^) [‘age’ F (2, 48) = 17.94, *P* < 0.0001; ‘genotype’ F (1, 24) = 61.34, *P* < 0.0001], volume (µm^3^) [‘age’ F (2, 48) = 8.320, *P* = 0.0008; ‘genotype’ F (1, 24) = 33.78, *P* < 0.0001], and perimeter (µm) [ ‘age’ F (2, 48) = 41.14, *P* < 0.0001; ‘genotype’ F (1, 24) = 97.21, *P* < 0.0001). However, the ‘age’ x ‘genotype’ interaction was only significant for convex 3D surface area (µm^2^) [F (2, 48) = 3.490, *P* = 0.0385] and convex perimeter (µm) [F (2, 48) = 5.034, *P* = 0.0104]. Post hoc test confirmed significant reduction in convex 3D surface area (Fig. 4M), in young (MD − 5694 ± 1431, ^**^p = 0.0030), and adult (MD − 8607 ± 1431, ^****^p < 0.0001) GFAP-IL6 mice compared to their age-matched control cohorts. There was also a significant reduction in surface area (µm^2^) within the aging cohorts between young and old control cohorts (MD -7916 ± 1431, ^####^*p* < 0.0001); adult and old control cohorts (MD − 6140 ± 1431, ^##^*p* = 0.0011); young and adult GFAP-IL6 cohorts (MD -4688 ± 1431, ^#^*p* = 0.0227); young and old GFAP-IL6 cohorts (MD − 5489 ± 1431, ^##^*p* = 0.0046). In terms of volume (Fig. 4N), we found a significant reduction in young (MD − 38,918 ± 11,251, ^*^p = 0.0138), and adult (MD − 56,168 ± 11,251, ^***^p = 0.0001) GFAP-IL6 mice compared to their age-matched controls. A significant reduction in volume within the aging cohorts were visible between young and old control cohorts (MD − 42,042 ± 11,251, ^##^*p* = 0.0062), and adult and old control cohorts (MD − 38,769 ± 11,251, ^#^*p* = 0.0143). Furthermore, in terms of perimeter (Fig. 4O), a significant reduction was found in young (MD − 81.11 ± 18.26, ^***^p = 0.0007), adult (MD − 140.1 ± 18.26, ^****^p < 0.0001), and old (MD − 61.32 ± 18.26, ^*^p = 0.0182) GFAP-IL6 cohorts compared to their age-matched control cohorts. There was also a significant reduction in perimeter (µm) within the aging cohorts between young and old control cohorts (MD − 144.5 ± 18.26, ^####^*p* < 0.0001); adult and old control cohorts (MD − 108.7 ± 18.26, ^####^*p* < 0.0001); young and adult GFAP-IL6 cohorts (MD − 94.75 ± 18.26, ^####^*p* < 0.0001); young and old GFAP-IL6 cohorts (MD − 124.7 ± 18.26, ^####^*p* < 0.0001).

**Fig. 5:**
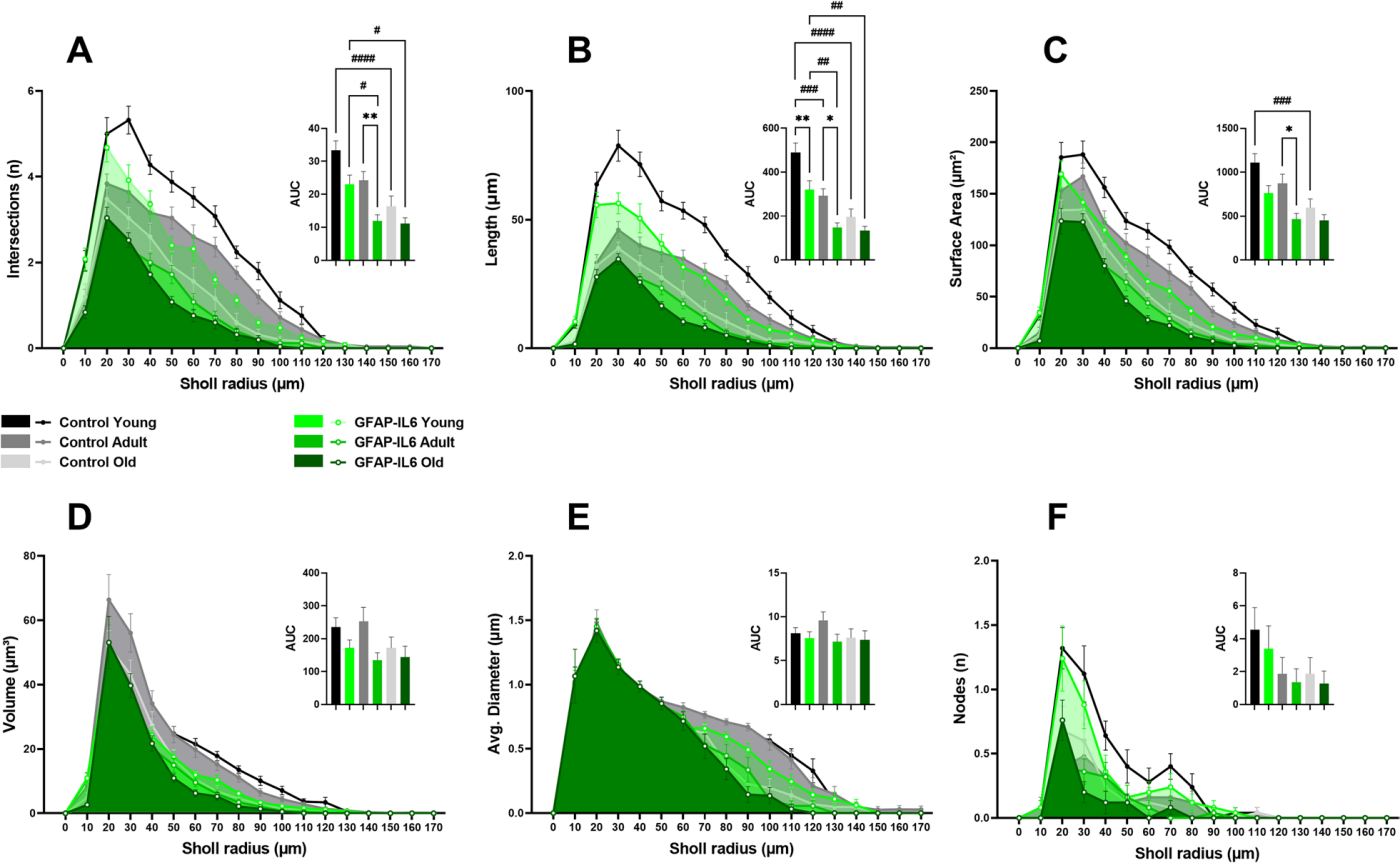
**A**–**F** Cumulative line graphs and corresponding area under the curve (AUC) bar graphs, representing the effects of aging and neuroinflammation on ChAT^+^ cholinergic cell dendritic arborization complexity in the medial septum (MS), measured by Sholl analysis. Quantitative analysis of individual ChAT^+^ cell dendritic arborization complexity including: **A** intersections (n), **B** length (µm), **C** surface area (µm^2^), **D** volume (µm^3^), **E** average diameter (µm), and **F** nodes (n) of young (*n = *25), adult (*n = *25) and old (*n = *25) mice from control and GFAP-IL6 cohorts. Data presented as mean ± SEM and analysed with two-way ANOVA followed by Tukey’s post hoc tests. Post hoc effects of ‘genotype’ are represented in asterisks (*p < 0.05, **p < 0.01, ***p < 0.001, ****p < 0.0001); post hoc effects of ‘age’ are represented by hash symbols (^#^*p* < 0.05, ^##^*p* < 0.01, ^###^*p* < 0.001, ^####^*p* < 0.0001)

Finally, we carried out the Sholl analysis on septal cholinergic neurons to study the changes in radial distribution of dendritic arborization from the cell soma, with aging and neuroinflammation. We observed less complex cholinergic dendritic arborization in GFAP-IL6 mice with aging, mostly confined to 0–80 µm radius, compared to age-matched control mice which showed interactions beyond the 90 µm radius (Fig. 5). After analysing AUC for each cumulative line graph for ChAT^+^ cells (Fig. 5A–F; Additional file 1: Supplementary Table S7), overall significant mean peak differences were observed between GFAP-IL6 mice and their age-matched controls for the measurements of intersections (n) [F (5, 2580) = 10.93, *P* < 0.0001], length (µm) [F (5, 2580) = 16.15, *P* < 0.0001], surface area (µm^2^) [F (5, 2580) = 8.290, *P* < 0.0001], and volume (µm^3^) [F (5, 2580) = 2.370, *P* = 0.0372]. No significant differences were observed among the cohorts for the average diameter (µm), and nodes (n). Post hoc comparisons displayed a significant reduction in AUC for intersections (Fig. 5A) in adult GFAP-IL6 cohort when compared to their age-matched control cohort (MD − 12.32 ± 3.633, ^**^p = 0.0092). A significant reduction was also observed among the aging cohorts between young and old control cohorts (MD − 16.88 ± 3.633, ^####^*p* < 0.0001); young and adult GFAP-IL6 cohorts (MD − 11.12 ± 3.633, ^#^*p* = 0.0271); young and old GFAP-IL6 cohorts (MD − 11.92 ± 3.633, ^#^*p* = 0.0134). Measurements of AUC length (Fig. 5B) displayed a significant reduction in young (MD − 167.7 ± 47.04, ^**^p = 0.0050), and adult (MD − 144.1 ± 47.04, ^*^p = 0.0268) GFAP-IL6 cohorts when compared to their age-matched control cohorts. A significant reduction was also observed among the aging cohorts between young and adult control cohorts (MD − 96.0 ± 47.04, ^###^*p* = 0.0005); young and old control cohorts (MD − 292.3 ± 47.04, ^####^*p* < 0.0001); young and adult GFAP-IL6 cohorts (MD − 172.4 ± 47.04, ^##^*p* = 0.0034); young and old GFAP-IL6 cohorts (MD − 186.9 ± 47.04, ^##^*p* = 0.0010). Finally, AUC measurements for surface area (Fig. 5C) displayed a significant reduction in adult GFAP-IL6 cohorts compared to age-matched control cohorts (MD − 406.1 ± 125.9, ^#^*p* = 0.0161). There was also a significant reduction seen between young and old control aging cohorts (MD − 511.9 ± 125.9, ^###^*p* = 0.0007) (see Additional file 1: Supplementary Table S8).

### Aging and chronic neuroinflammation are associated with degenerating fibres in the medial septum

**Fig. 6:**
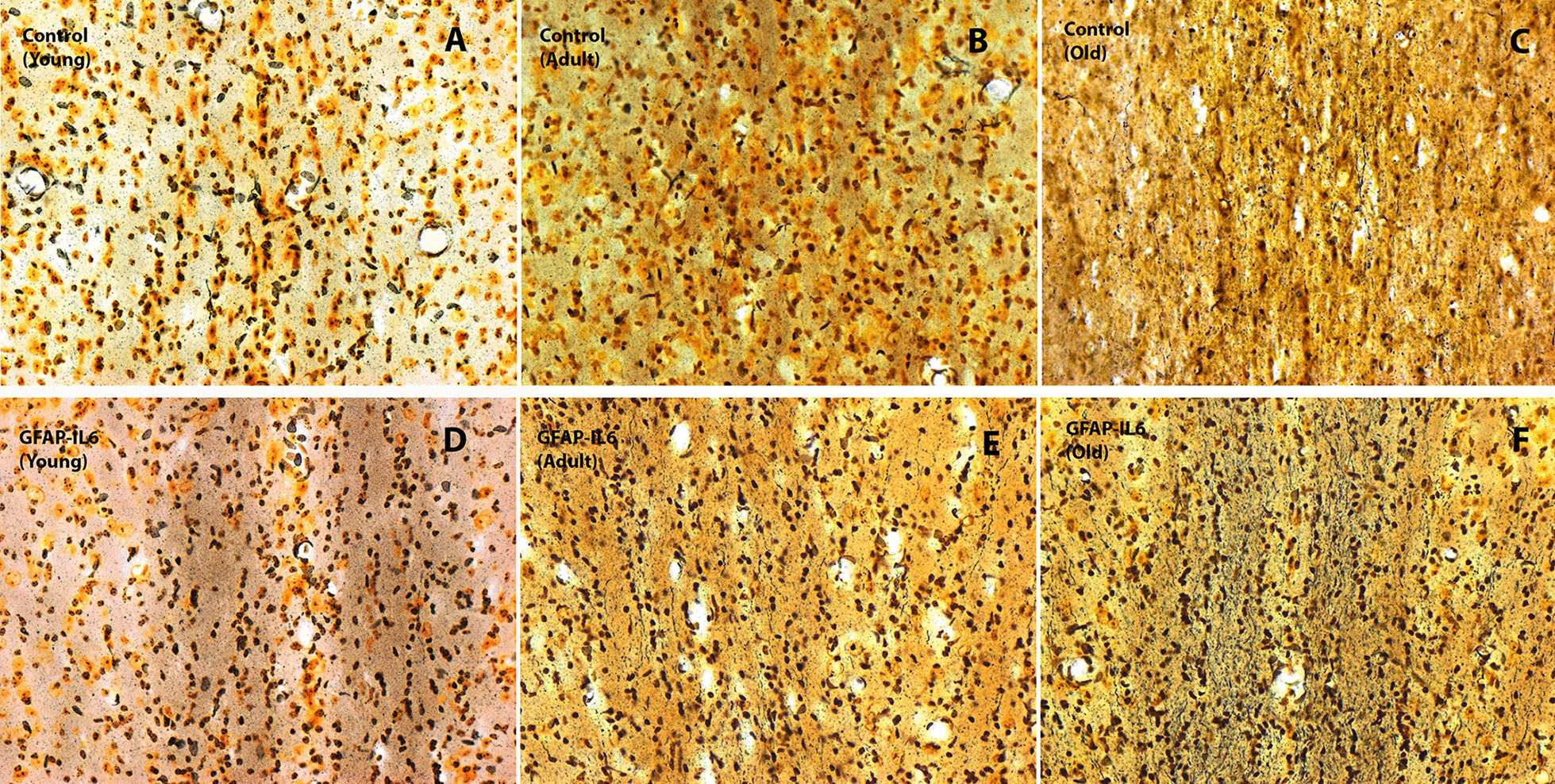
**A–F** Representative bright-field microscopy images revealing degenerating fibres in the medial septum (MS) during aging and neuroinflammation. While background silver-staining of nuclei was visible in all sections, silver-stained fibres were not prominently observed in the sections from **A** young control mice compared to much aged control mice **B** adult and **C** old. The GFAP-IL6 mice **D–F** presented with darkly stained fibres at all three time points, with highest intensity staining in old mice. Images taken under the 20 × objective (scale bar 40 µm)

To further investigate the medial septal cholinergic degenerating axons due to aging and chronic neuroinflammation, we performed a silver staining for degenerating cells to assess the medial septum. Background silver-staining of nuclei was evident in all sections. However, silver-stained fibres were not prominently observed in the sections from young control cohort compared to adult and old control cohorts (Fig. 6A–C). Moreover, the GFAP-IL6 cohorts exhibited darkly stained fibres at all three time points, with highest intensity staining in the old cohort (Fig. 6D–F).

### Aging and chronic neuroinflammation are associated with pyramidal dendritic spine loss in the hippocampus

**Fig. 7:**
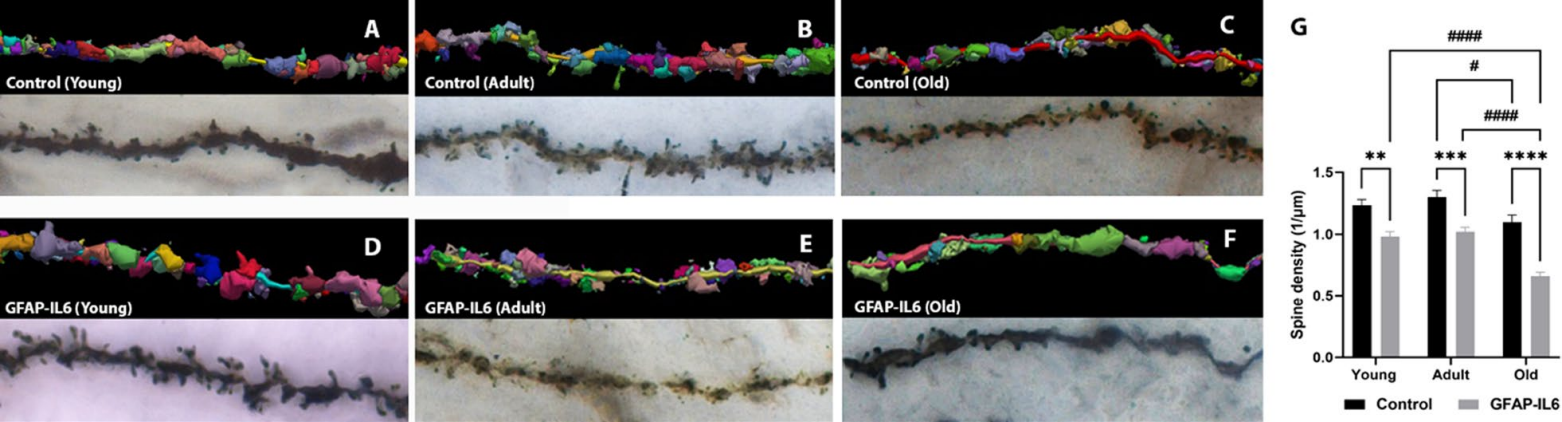
A-G Representative images of reconstructed hippocampal pyramidal spines revealing changes during aging and neuroinflammation. The bright-field Golgi-stained images of reconstructed pyramidal spines (**A**–**F**) represent changes in young, adult, and old mice from control and GFAP-IL6 cohorts. The grouped bar graph (**G**) represents the estimated spine density per µm of reconstructed pyramidal dendrite in the hippocampus of young (*n = *20), adult (*n = *20) and old (*n = *20) mice from control and GFAP-IL6 cohorts. Data presented as mean ± SEM and analysed with two-way ANOVA followed by Tukey’s post hoc tests. Post hoc effects of ‘genotype’ are represented in asterisks (*p < 0.05, **p < 0.01, ***p < 0.001, ****p < 0.0001); post hoc effects of ‘age’ are represented by hash symbols (^#^*p* < 0.05, ^##^*p* < 0.01, ^###^*p* < 0.001, ^####^*p* < 0.0001). Images taken under the 60× oil-objective (scale bar 100 µm)

To investigate if the changes observed within the septal ChAT^+^ cholinergic cell population have reciprocal effects on the hippocampal pyramidal neuronal spine density, we performed a spine density analysis (Fig. 7A–F). A main effect of ‘age’ and ‘genotype’ was observed in the measurements of spine density (1/µm), with no ‘age’ x ‘genotype’ interaction [‘age’ F (2, 138) = 21.90, *P* < 0.0001; ‘genotype’ F (1, 138) = 76.04, *P* < 0.0001]. Post hoc test confirmed a significant reduction in spine density (Fig. 7G) in young (MD − 0.2477 ± 0.06388, ^**^p = 0.0022), adult (MD − 1.020 ± 0.2787, ^***^p = 0.0004), and old (MD − 0.4384 ± 0.06388, ^****^p < 0.0001) GFAP-IL6 mice when compared to their age-matched control cohorts. Furthermore, spine density significantly reduced within the aging cohorts between adult and old control cohorts (MD − 0.2014 ± 0.06388, ^#^*p* = 0.0238); young and old GFAP-IL6 cohorts (MD − 0.3235 ± 0.06388, ^####^*p* < 0.0001); adult and old GFAP-IL6 cohorts (MD − 0.3611 ± 0.06388, ^####^*p* < 0.0001).

### The impact of aging and neuroinflammation on the intrinsic passive properties of cholinergic neurons in the MS

**Fig. 8:**
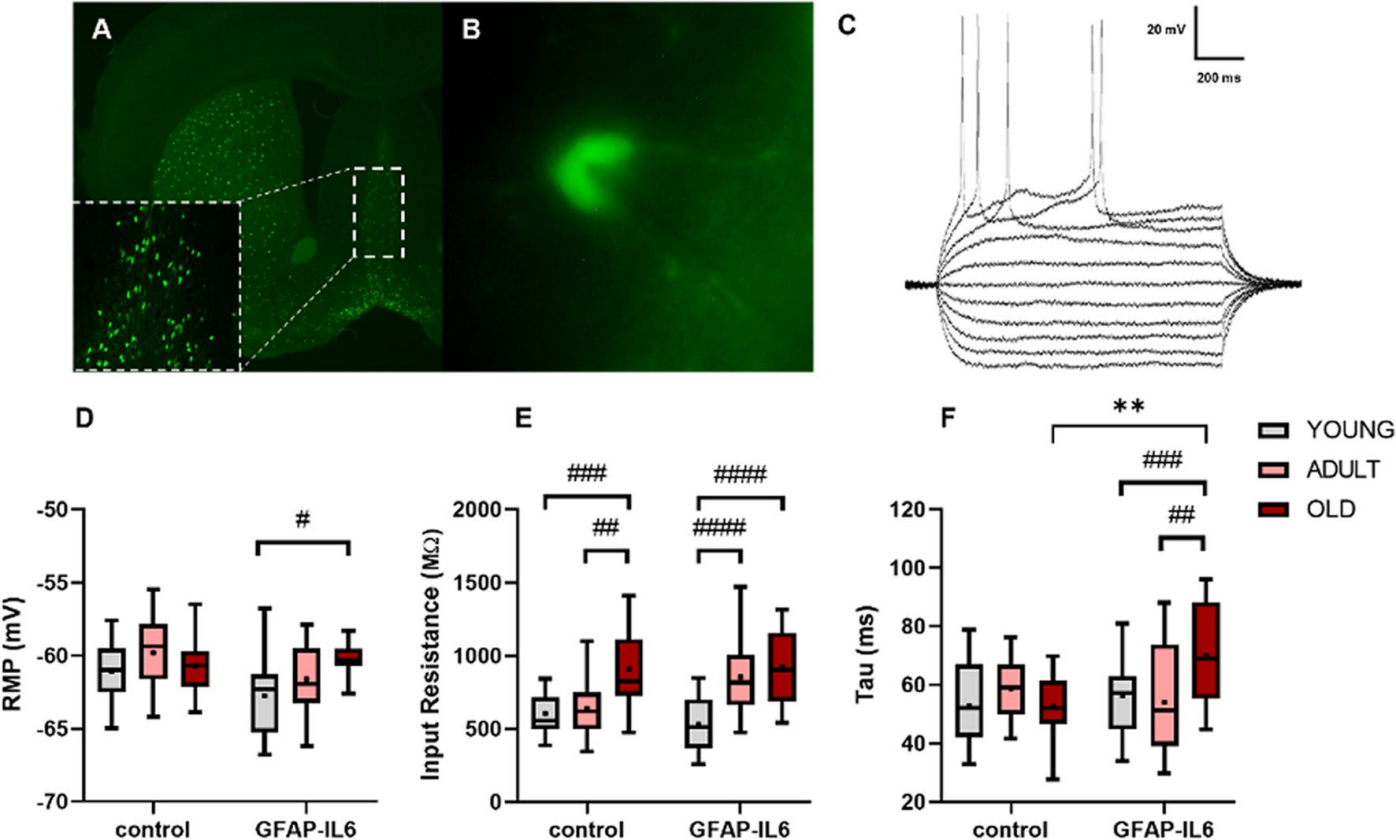
The impact of aging and chronic neuroinflammation on the passive membrane properties of cholinergic neurons in the MS. **A** Representative confocal image depicting the MS in control mouse obtained under 2.5× magnification. Inset−10 × magnification of the MS. **B** Representative picture (× 40) depicting a cholinergic neuron with the recording electrode during whole-cell patch-clamp. **C** Sample traces of electrophysiological membrane properties in response to depolarizing and hyperpolarizing current injections. **D**–**F** Box plots depicting the intrinsic passive properties of the cholinergic neurons in the MS of control and GFAP-IL6 mice at young (3–4 months, light grey), adults (9–10 months, pink), and old (18–19 months, dark red) groups. The properties of resting membrane potential (**D**), input resistance (**E**), and time constant tau (**F**). Data are shown as box plot, the box upper and lower limits are the 25th and 75th quartiles, respectively. The whiskers depict the lowest and highest data points, the horizontal line through the box is the median and the + sign represents the mean, two-way ANOVA, with Tukey’s post hoc test. Post hoc effect of ‘genotype’ indicated by asterisks, (*p < 0.05, **p < 0.01, ***p < 0.001, ****p < 0.0001); post hoc effect of ‘age’ indicated by hash symbols (#*p* < 0.05, ##*p* < 0.01, ###*p* < 0.001, ####*p* < 0.0001)

To assess alterations in the intrinsic electrophysiological properties of cholinergic neurons during aging and chronic neuroinflammation, we have recorded both passive (resting membrane potential (RMP), input resistance (Rin), membrane time constant-(tau)) and active membrane properties of BFCN neurons in the MS of control and GFAP-IL6 mice **(**Fig. 8A–C**)**. Our results suggest that the RMP was affected by both aging and chronic neuroinflammation (*F*_*(2,108)*_ = 4.32, *p* < 0.05 for the factor aging; *F*_*(1,108)*_ = 6.26, *p* < 0.01 for the factor chronic neuroinflammation, and *F*_*(2,108)*_ = 2.88, *p* = 0.06 for the factor interaction, two-way ANOVA; Fig. 8E). However, while the RMP in ChAT mice was comparable between age groups (*p* > 0.05, two-way ANOVA, Fig. 1D), it depolarized throughout aging in IL6-ChAT mice (− 62.76 ± 0.65 mV, *n = *19 in young mice vs. − 60.35 ± 0.26 mV, *n = *20 in aged mice, *p* < 0.05, two-way ANOVA with Tukey’s post hoc test), indicating that the aging process itself in control mice is not sufficient to induce changes in the RMP. In addition, aging alone and combined with chronic inflammation had a significant impact on the input resistance (F_(2,112)_ = 24.29, *p* < 0.0001 for the factor aging; F_(1,112)_ = 1.68, *p* = 0.2 for the factor chronic neuroinflammation; F_(2,112)_ = 4.84, *p* = 0.01 for the factor interaction, two-way ANOVA). Post hoc analysis indicated that the average input resistance significantly increased with age in the ChAT mice (604.8 ± 31.99 MΩ, *n = *18 in Young mice vs 908.8 ± 65.06 MΩ, *n = *17, in aged mice *p* < 0.001 and adult 638.5 ± 40.77 MΩ, *n = *21; *p* < 0.01, two-way ANOVA with Tukey’s post hoc test, Fig. 8E). Similarly, the young IL6-ChAT mice had a lower input resistance (531.4 ± 41.77 MΩ, *n = *20) compared to both the adult (853.7 ± 49.85 MΩ, *n = *22, *p* < 0.0001) and aged group (921.6 ± 56.48 MΩ, *n = *20, *p* < 0.0001, two-way ANOVA with Tukey’s post hoc test, Fig. 8E).

Similarly, chronic neuroinflammation during aging had significant impact on the membrane time constant (F_(2,116)_ = 2.31, *p* = 0.1 for the factor aging; F_(1,116)_ = 4.1, *p* = 0.05 for the factor chronic neuroinflammation; F (_2,116)_ = 5.99, *p* = 0.003 for the factor interaction, two-way ANOVA). Post hoc analysis indicated that the average tau in the aged L6-ChAT mice (69.95 ± 3.73 ms, *n = *20) was significantly higher than both adults (54.02 ± 4.01 ms, *n = *22, *p* < 0.01, two-way ANOVA with Tukey’s post hoc test) and young mice (56.18 ± 2.78 ms, *n = *22, *p* < 0.05, two-way ANOVA with Tukey’s post hoc test, Fig. 8F). While the tau in ChAT mice was comparable between age groups (*p* > 0.05, two-way ANOVA, Fig. 8F), the aged group showed an increase between ChAT and IL6-ChAT (aged ChAT 52.69 ± 2.79 ms, *n = *17; aged IL6-ChAT 69.95 ± 3.73 ms, *n = *20, **p < 0.01, two-way ANOVA with Tukey’s post hoc test) indicating that this age-group is specifically susceptible for chronic neuroinflammation. These results highlight the impact of chronic neuroinflammation on the passive membrane properties of BFCNs at different age-groups, specifically the adult and aged groups that express an increased excitability profile.

### The impact of aging and neuroinflammation on the active membrane properties of cholinergic neurons in the MS

**Fig. 9:**
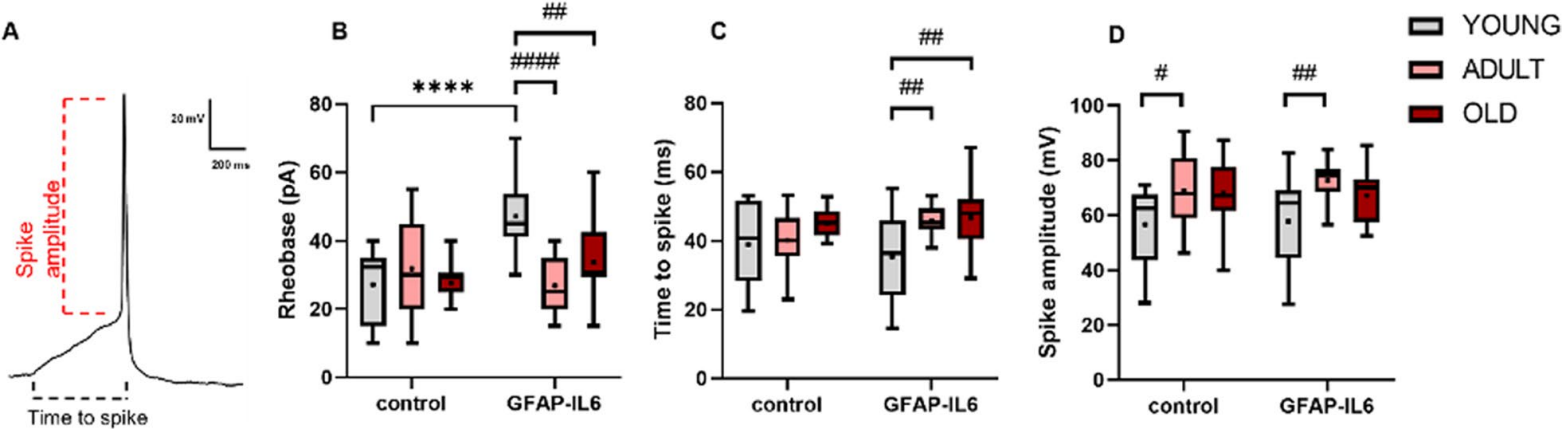
The impact of aging and chronic neuroinflammation on the active membrane properties of cholinergic neurons in the MS. **A** Example action potential trace, detailing the parameters of spike amplitude and time to spike. **B**–**D** Box plots depicting the intrinsic active properties of the cholinergic neurons in the MS of control and GFAP-IL6 mice at young (light grey), adults (pink), and old (dark red) groups. The properties shown are rheobase (**B**), time to spike (**C**), spike amplitude (**D**). Data are shown as box plot, the box upper and lower limits are the 25th and 75th quartiles, respectively. The whiskers depict the lowest and highest data points, the horizontal line through the box is the median and the + sign represents the mean, two-way ANOVA, with Tukey’s post hoc test. Post hoc effect of ‘genotype’ indicated by asterisks, (*p < 0.05, **p < 0.01, ***p < 0.001, ****p < 0.0001); post hoc effect of ‘age’ indicated by hash symbols (#*p* < 0.05, ##*p* < 0.01, ###*p* < 0.001, ####*p* < 0.0001)

To further understand the impact of aging and neuroinflammation on the excitability profile of BCFN neurons, we recorded their active membrane properties, including spike rheobase (the minimum current required to elicit an action potential), time to spike and spike amplitude (Fig. 9A). Aging had an impact on all the active membrane properties. Two-way ANOVA analysis for spike rheobase showed an impact factor for aging equal to F(2,101) = 5.23, *p* = 0.007; time to spike had a significant value of F(2,110) = 8.76, *p* = 0.0003; and the spike amplitude was significant as well, F(2,111) = 13.39, *p* < 0.0001 (Fig. 9B–D). Chronic neuroinflammation significantly affected the spike rheobase F(1,101) = 11.89, *p* = 0.0008, but not the time to spike (F(1,110) = 0.47, *p* = 0.49, two-way ANOVA) or the spike amplitude (F(1,111) = 0.35, *p* = 0.55, two-way ANOVA). Similarly, the interaction factor between aging and chronic neuroinflammation was significant for the spike rheobase (F(2,101) = 12.61, *p* < 0.0001, two-way ANOVA) but not for the time to spike (F(2,110) = 2.45, *p* = 0.09, two-way ANOVA) and spike amplitude (F(2,111) = 0.37, *p* = 0.069, two-way ANOVA).

The analysis of the spike rheobase revealed significant differences in the GFAP-IL6 mice, as in the old (33.75 ± 2.46 pA, *n = *20, ^##^*p* < 0.01, two-way ANOVA with Tukey’s post hoc test) and in the adult groups (26.90 ± 1.6 pA, *n = *21, ^####^*p* < 0.0001, two-way ANOVA with Tukey’s post hoc test) the rheobase current decreased compared to the young GFAP-IL6 (47.19 ± 2.67 pA, *n = *16), with the lowest current amplitude reached by the adult group (Fig. 9B). Post hoc analysis indicated that in the adult age group, the spike amplitude (Fig. 9D) increased in both genotypes compared to the young group [(control adult, 68.77 ± 3 mV, *n = *21; control young, 56.71 ± 2.97 mV, *n = *19, ^#^*p* < 0.05, two-way ANOVA with Tukey’s post hoc test); (GFAP-IL6 adult, 72.62 ± 1.8 mV, *n = *20; GFAP-IL6 young, 57.81 ± 3.43 mV, *n = *21, ^##^*p* < 0.01, two-way ANOVA with Tukey’s post hoc test)]. These results indicate an increase in the excitability profile of the old and adult GFAP-IL6, which is also associated with an increase in the time to spike compared to the young group **(**Fig. 9C) (GFAP-IL6 young, 35.37 ± 2.87 ms, *n = *21; GFAP-IL6 adult, 45.73 ± 0.94 ms, *n = *19, ^##^*p* < 0.01; GFAP-IL6 old, 46.63 ± 2 ms, *n = *20, ^##^*p* < 0.01, two-way ANOVA with Tukey’s post hoc test).

**Fig. 10:**
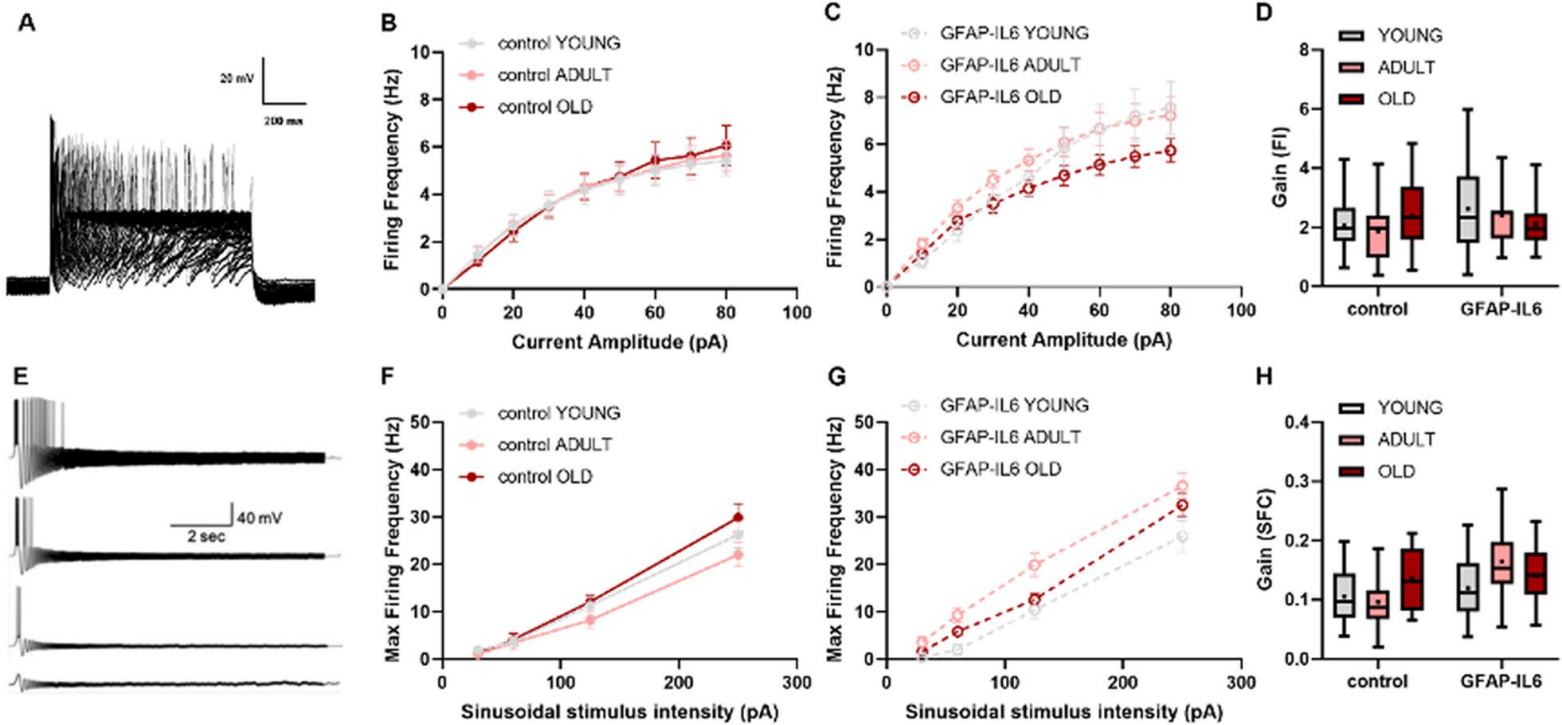
The effect of aging and chronic neuroinflammation on the excitability profile of cholinergic neurons in the MS. **A** Sample voltage traces recorded after step current injections from 10 pA to 300 pA representing the firing frequency–current relationship (F–I curve). **B, C** Graphs of the F–I curves for all the groups describing the relationship between the current injected into cholinergic neurons and the firing frequency associated with the stimulus. The upward shift of the F–I curve in the young and adult group of IL6-control is indicative of increased excitability. **D** Box plot depicting the average gain of the F–I curves in all groups tested. **E** Sample voltage traces recorded following sinusoidal chirp stimulation (0.1–100 Hz) at different intensities (from bottom to top: 30 pA, 60 pA, 125 pA, 250 pA). **F**, **G** Graphs of the SFC curves for all the groups describing the relationship between the oscillation intensity and the maximal frequency at which the cell is still excitable. Note the upward shift in the maximal oscillation frequency in adult GFAP-IL6 mice, indicative of increased responsiveness compared to the age-matched control group. **H** B*ox* plot depicting the average gain of the SFC curves in all groups tested. Data are shown as box plot, the box upper and lower limits are the 25th and 75th quartiles, respectively. The whiskers depict the lowest and highest data points, the horizontal line through the box is the median and the + sign represents the mean, two-way ANOVA, with Tukey’s post hoc test. Post hoc indicated by asterisks (*p < 0.05, **p < 0.01, ***p < 0.001, ****p < 0.0001)

Neuronal firing properties are highly dependent on their excitability profile and reflect the dynamic interplay between their passive and active characteristics (Fig. 10). We therefore assessed the impact of chronic neuroinflammation and aging on the firing patterns of cholinergic neurons in the MS. To this extent, we measured the firing frequency–current relationship (F–I curves) following injections of increasing step currents, as illustrated in (Fig. 10A). Our results indicate comparable gains between all age groups of control mice, suggesting that aging itself does not affect the firing properties (Fig. 10B, C). The gain of the F–I curve showed a tendency to increase in the young (2.63 ± 0.33, *n = *21) and adult (2.42 ± 0.2, *n = *20) GFAP-IL6 mice compared to their age-matched control mice (young control, 2.06 ± 0.2, *n = *21; adult control, 1.88 ± 0.22, *n = *21 (Fig. 10D). Under physiological conditions, neuronal firing patterns are embedded within network oscillations termed brain waves. In order to assess the firing patterns of BCFN neurons under oscillatory regimes, we measured the relationship between membrane oscillation frequencies and spike threshold through injection of sinusoidal currents at increasing frequencies (0.1–100 Hz) (Fig. 10E) and different intensities (30–250 pA chirp current). This protocol allows an evaluation of the relationship between neuronal excitability and oscillatory behaviour, depicted by the frequency spiking curve (SFC), as we previously described [59]. Comparison between SFC’s recorded in control mice indicated an age-dependent biphasic pattern, in which neurons from adult mice show a decrease in the max firing frequency under high stimulation (250 pA) (Fig. 10F, G), which increased in old mice. However, a comparison between the oscillatory activity in control and IL-6 mice indicated a significant increase of the SFC’s gain only in the adult age group (Fig. 10H). Our data show that following 60, 125, and 250 pA chirp stimulation, cholinergic neurons of the GFAP-IL6 adult group are capable of firing action potentials at higher oscillation frequencies compared to the adult control mice and overall are more excitable than their age-matched controls (gain of SFC adult control, 0.096 ± 0.01, *n = *22,; adult GFAP-IL6, 0.16 ± 0.01, *n = *26, ***p < 0.001, two-way ANOVA with Tukey’s post hoc test (Fig. 10H). These results suggest that chronic neuroinflammation effects the excitability profile of cholinergic neurons in the MS, specifically the adult age group.

## Discussion

In this study, we investigate the accumulating effect of aging and chronic microglial activation on the medial septal cholinergic cell population for the first time, using the GFAP-IL6 mouse model. We found a significant increase in septal Iba-1^+^ microglia number and density with normal aging in control mice, which was exacerbated with continuous IL-6 expression in GFAP-IL6 mice. In contrast, the septal ChAT^+^ cholinergic cell number and density were not significantly reduced among the normal aging cohorts. Our findings align with previous studies conducted to quantified cholinergic neurons and activated microglia (depicted as activated Iba-1^+^ or CD68^+^ microglia) in the MS/VDB of young (3–6 months) and old (24–28 months) Fisher 344 × Brown Norway F1 rats [60, 61]. A similar observation was reported for 7, 5, and 53-month old C57BI/6NNIA mice, stating comparable estimates of the total number of cholinergic neurons in the MS [62]. Hence, according to these previous reports, aging is characterized by an increase in the basal inflammatory state within the MS/VDB, but this inflammation does not correlate with cholinergic neuron death, or the loss of cholinergic neurons does not necessarily link to impaired spatial learning in aged rats [60, 61].

A previous study that used lipopolysaccharide (LPS) to produce acute inflammation within the BF of young rats (3 months old) showed that acute exposure to LPS decreased cortical ChAT activity and the number of ChAT-positive cells within the BF, along with a dense distribution of reactive astrocytes and microglia [63]. Our group has previously reported significant activation/priming of microglia in the medial septum following LPS (500 μg/kg) injection in ChAT^BAC^-eGFP mice, as well as age-related biphasic excitability profile of cholinergic neurons with increased excitability at adulthood (ages 9 and 12 months) and decreased excitability in aged animals (> 18 months) [33]. Most importantly, our findings from the current study for the first time present a significant reduction in the septal ChAT^+^ cell number in adult (12-month) and old (24-month) GFAP-IL6 mice, while the cholinergic cell density was significantly reduced only in the young GFAP-IL6 mice of 3-month age compared to the age-matched control mice. This suggests that pathological aging with brain-specific chronic IL-6 expression in GFAP-IL6 mice, might have detrimental effects on the septal ChAT^+^ cholinergic population compared to the effect of normal aging, and it might underlie the genesis of some neuropathological changes associated with age-related progressive neurodegenerative diseases like AD.

Sex differences in mice were not taken into account in the current study because previous studies have reported no difference in the number of ChAT^+^ BFCNs between genotype-matched males and females in the MS/VDB and HDB [64].

In addition, it is noteworthy that the absolute number of ChAT^+^ septal cholinergic cells in the GFAP-IL6 animals did not change significantly during the aging process. Since in this mouse model IL-6 is released by astrocytes in utero from the formation of the astroglia cells, it is possible that cholinergic cell formation during the embryonic development is already impacted [65–67]. Additionally, chronic IL-6 pathology at a young age would further hinder or delay the development of the cholinergic system and dysregulate compensatory mechanisms [68]. This could be the reason for limited change in septal cholinergic cell density in adult and aged mice regardless of pathology. Moreover, lower septal cholinergic cell number and density in the young 3-month aged mice compared to older mice could be an indication of the cholinergic system developing a compensatory upregulation of cholinergic markers in older age [69–71]. It should also be noted that the cholinergic neuron density is not uniform across different brain regions, even within the same brain nucleus [72].

Complimenting these findings, we observed a significant reduction in septal volume in adult and aged GFAP-IL6 mice compared to age-matched control mice. This reduction in septal volume was significant in adult and aged GFAP-IL6 mice compared to the young GFAP-IL6 mice, indicating that septal volume is barely affected by normal aging, but largely affected by brain-specific chronic IL-6 expression which could further worsen with aging among the GFAP-IL6 mice. A recent study using in vivo MRI in 3xTg AD mice revealed that MS atrophy significantly reduced cholinergic neuron number, but this does not necessarily contribute to overall septal volume loss, and thus volume change cannot be taken as a direct biomarker of cholinergic neuron number [73]. However, in this study, the MS was contoured bordering the ChAT^+^ cell distribution, and the MS volume was explicitly defined by the ChAT^+^ cholinergic cell number. Hence, when the ChAT^+^ cholinergic cell number was significantly reduced among the adult and old GFAP-IL6 mice, we also observed a corresponding volume loss, and as a result the ChAT^+^ cell density was not significantly changed. Moreover, because there was no change in septal volume, but ChAT^+^ cell number displayed a tendency to decrease in young GFAP-IL6 mice compared to age-matched control, the change in density was significant only at this age group.

As previously reported, Iba-1^+^ microglia of the GFAP-IL6 cohorts were found to have morphological features resembling reactive ‘de-ramified’ microglia (retracted thick processes, enlarged cell soma, and decreased arborization), in comparison to the surveillant ‘ramified’ morphology (motile fine processes, small cell soma, and distal arborization) observed in the control cohorts [8, 35, 36, 38]. We observed significant differences in septal Iba-1^+^ microglia population with both normal aging and chronic IL-6 expression. Branched structure analysis, for aging GFAP-IL6 mice compared to age-matched control mice, displayed significant increase in soma area and perimeter, while exhibiting a reduction in circularity, total process length and nodes. The prominent morphological re-orientation of these septal microglia towards a re-activated and de-ramified state, characterized by short and thick branch processes with enlarged soma, represent a neurotoxic phenotype contributing to neurodegeneration [74–77]. These observations were further confirmed by overall reduction in septal Iba-1^+^ microglia spatial (convex-hull parameters), and ramification (Sholl parameters) complexity among the aging GFAP-IL6 mice compared to age-matched control mice. Overall, indicating that aging together with neuroinflammation could significantly affect the morphology of the septal Iba-1^+^ microglia population, possibly leading to detrimental neurodegenerative effects.

Our analysis of septal ChAT^+^ cholinergic cell morphology indicated neurodegenerative phenotype in adult and old GFAP-IL6 mice compared to age-matched control mice. In terms of branch structure, neuroinflammation seemed to cause slight swelling of the septal ChAT^+^ cholinergic cell soma, resulting in an increase in soma area, perimeter, and circularity was observed among GFAP-IL6 mice compared to age-matched control mice. This could be a result of various reactive changes due to retrograde damage toward the neuronal cell soma, particularly following axonal damage, and the resultant neuronal swelling (chromatolysis) associated with increased protein synthesis to accommodate for axonal sprouting [78, 79]. Moreover, it is known that cholinergic neurons are among the most energy-consuming neurons, because they require supplementary production of acetyl-CoA by their mitochondria, thus making them highly sensitive to toxicity from excess microglial activation during inflammation, and also to oxidative stress during aging [25, 80]. Aligning with these observations, processes from the soma, total length of dendrites, and nodes displayed a significant reduction in adult and old GFAP-IL6 mice compared to young GFAP-IL6 mice and age-matched control mice. The reduction in the total length of dendrites, and nodes was also significant among young and adult as well as young and old control mice, thus showing an effect of aging on the cholinergic cell population. The spatial dendritic arborization complexity in terms of surface area, volume, and perimeter significantly reduced among the young and old mice of both GFAP-IL6 and control aging mice, while GFAP-IL6 mice displayed much exacerbated degenerative characteristics compared to their age-matched control cohorts. The Sholl parameters for intersections, length, and surface area further confirmed these degenerative changes. We further observed that these changes in morphological parameters are stabilizing, and not displaying significant changes between adult and old GFAP-IL6 mice, indicating that continuous IL-6 expression that persists through adulthood could lead to detrimental changes to neuronal morphology leading to similar progressive neurodegenerative changes seen in aged individuals. Previous studies have shown progressive age-related decline in BFCN cell size both in normal aging and in AD mouse models [62].

Degenerative changes in the mouse MS with aging and neuroinflammation was further confirmed qualitatively using the histological silver-staining method for degenerating cells. This method is based on the principle that in neurons undergoing degeneration, certain cellular components such as lysosomes, axons, and terminals become argyrophilic (readily stained black by silver salts) and bind to silver ions with high affinity [79]. These bound silver ions, upon reduction, form metallic grains that are visible under a light or an electron microscope, and is proven to be very specific and sensitive for the detection of degenerating neurons in the CNS [79, 81]. We observed distinct degenerative features (degenerating dendrites and axon terminals) in the MS of adult and old GFAP-IL6 mice compared to their age-matched controls, representing various degrees of disintegration visible as selective black impregnation in the bright field image panel.

Coinciding with our data obtained for septal ChAT^+^ cholinergic cell number and morphology, there was a significant reduction in CA1 hippocampal pyramidal dendritic spine density in young, adult, and old GFAP-IL6 mice compared to age-matched control mice. Furthermore, this reduction in spine density was significant among aging cohorts between young and old GFAP-IL mice; adult and old GFAP-IL6 mice; adult and old control mice. These reductions in spine density could be a result of the possible loss or degeneration of septal cholinergic projection neurons that synapses onto hippocampal CA1 pyramidal neurons. Aligning with our findings, a previous study on young (3-month) and adult (24-month) rats found a significant reductions in cholinergic axonal length and in synaptophysin protein levels in the aged hippocampus [61]. A previous diffusion MRI (dMRI) study reported that after causing selective BFCN degeneration in mice via the toxin saporin conjugated to a p75 neurotrophin receptor antibody (mu-p75-SAP), it resulted in ~ 25% loss of the BFCN population and significant loss of terminal cholinergic projections in the hippocampus [82]. Furthermore, another dMRI study using the 3xTg-AD mouse model at 2, 8, and 15 months of age, presented age-related hippocampal cholinergic neuritic dystrophy and accompanied white matter disruption in the septo-hippocampal pathway [83].

Future studies should aim to assess the functional properties of the septal cholinergic neurons in aging GFAP-IL6 mice together with changes in hippocampal CA1 pyramidal cell firing patterns using synaptic theta-burst stimulation (TBS) [84]. This will help in understanding the importance of cholinergic input in modulating hippocampal activity and the resultant dysfunctions in pathological aging. These findings could also be extended to access the behavioural phenotype in GFAP-IL6 mice related to cognition and hippocampal plasticity, using appropriate behavioural tests such as the open-field test and Morris water maze (MWM). Future studies should also aim to dissect the effect of continuous IL-6 expression in other BFCN populations, and changes in other pro-inflammatory markers such as Triggering Receptor Expressed on Myeloid Cells 2-DNAX activation protein 12 (TREM2-DAP12) TREM-2 expressed exclusively in microglia [85].

## Conclusion

In conclusion, this study assessed the effect of normal aging and neuroinflammation on the medial septal Iba-1^+^ microglia population and the consequential effect on the ChAT^+^ cholinergic cell population. Chronic IL-6 expression in the mouse brain significantly increased the reactive Iba-1^+^ microglia and decreased the ChAT^+^ cholinergic cell number in the medial septum and this tendency exacerbated with aging. We also showed that aging and chronic IL-6 expression reorientated the septal microglia morphology towards a more reactive and de-ramified pro-inflammatory phenotype. These findings further reflected upon the septal cholinergic cell population displaying neurodegenerative phenotype. The resultant direct effect of neuroinflammation and aging on the septal cholinergic population mirrored upon the hippocampal pyramidal dendritic spine density. Overall, these findings demonstrate a potential detrimental effect of chronic microglia activation on the MS, which can exacerbate throughout aging, leading to cholinergic dysfunction that could in turn disrupt hippocampal pyramidal cell network regulation. Hence, indicating that the GFAP-IL6 mouse model is suitable to study neuroanatomical changes in age-related progressive neurodegenerative diseases with an inflammatory component.

### Supplementary Information

**Additional file 1: Table S1.** Summary of stereological parameters used to count Iba-1^+^ microglia in the medial septum and septal volume results. **Table S2.** Summary of stereological parameters used to count ChAT^+^ cholinergic cells in the medial septum and septal volume results. **Table S3.** Summary of stereological data for Iba-1^+^ microglia and ChAT^+^ cholinergic cells in the mouse medial septum. **Table S4.** Summary of morphological analyses results for medial septal Iba-1^+^ microglia. **Table S5.** Summary of sholl analyses, area under the curve (AUC), results for medial septal Iba-1^+^ microglia. **Table S6.** Summary of morphological analyses results for medial septal ChAT^+^ cholinergic cells. **Table S7.** Summary of sholl analyses, area under the curve (AUC), results for medial septal ChAT^+^ cholinergic cells. **Table S8.** Summary of hippocampal pyramidal neuronal spine density analysis.

## Data Availability

The original contributions presented in the study are included in the article/additional file, further inquiries can be directed to the corresponding author/s.

## Abbreviations

AD: Alzheimer’s disease
BFCN: Basal forebrain cholinergic neuron
MS: Medial septum
BF: Basal forebrain
VP: Ventral pallidum
VDB: Vertical diagonal band nuclei
HDB: Horizontal diagonal band nuclei
SI/EA: Substantia innominata/extended amygdala
ChAT: Choline acetyl transferase
CA1: Cornu ammonis 1 region
REM: Rapid eye movement sleep
ACh: Acetylcholine
AChE: Enzyme acetylcholinesterase
CNS: Central nervous system
Aβ: Β-amyloid
IL-6: Interleukin-6
GFAP-IL6: Glial fibrillary acidic protein-interleukin 6
BAC: Bacterial artificial chromosome
EGFP: Enhanced green fluorescent protein
LPS: Lipopolysaccharide
TBS: Theta-burst stimulation
MWM: Morris water maze
TREM2-DAP12: Triggering receptor expressed on myeloid cells 2-DNAX activation protein 12
