# Supplementary material for: Chronic neuroinflammation during aging leads to cholinergic neurodegeneration in the mouse medial septum
Source: J Neuroinflammation. 2023 Oct 13;20:235. doi: 10.1186/s12974-023-02897-5 (PMC10576363; doi:10.1186/s12974-023-02897-5)
Supplement: Supplementary file 1 — Additional file 1: Table S1. Summary of stereological parameters used to count Iba-1+ microglia in the medial septum and septal volume results. Table S2. Summary of stereological parameters used to count ChAT+ cholinergic cells in the medial septum and septal volume results. Table S3. Summary of stereological data for Iba-1+ microglia and ChAT+ cholinergic cells in the mouse medial septum. Table S4. Summary of morphological analyses results for medial septal Iba-1+ microglia. Table S5. Summary of sholl analyses, area under the curve (AUC), results for medial septal Iba-1+ microglia. Table S6. Summary of morphological analyses results for medial septal ChAT+ cholinergic cells. Table S7. Summary of sholl analyses, area under the curve (AUC), results for medial septal ChAT+ cholinergic cells. Table S8. Summary of hippocampal pyramidal neuronal spine density analysis. [file 12974_2023_2897_MOESM1_ESM.docx]

Table 1. Summary of stereological parameters used to count Iba-1^+^ microglia in the medial septum and septal volume results.

| **Data set** | **Counting frame size (xy) µm^2^** | **Grid size (xy) µm^2^** | **Number of sections** | **Dissector height (µm)** | **Distance between sections (µm)** | **Number of sampling sites** | **Number of Iba-1^+^ cells counted** | **Gunderson coefficient error (m=1)** | **Estimated number of Iba-1^+^ cells** | **Measured Volume (mm³)** | **Density n/mm^3^** |
| --- | --- | --- | --- | --- | --- | --- | --- | --- | --- | --- | --- |
| ***Young Control*** | | | | | | | | | | | |
| 3433 | 120x120 | 220x220 | 7 | 35 | 150 | 211 | 214 | 0.07 | 3333.74 | 0.44 | 7499.71 |
| 3513 | 120x120 | 220x220 | 7 | 35 | 150 | 212 | 235 | 0.07 | 3835.97 | 0.40 | 9607.39 |
| 3993 | 120x120 | 220x220 | 7 | 35 | 150 | 178 | 225 | 0.07 | 3883.44 | 0.45 | 8704.43 |
| 3994 | 120x120 | 220x220 | 7 | 35 | 150 | 222 | 258 | 0.06 | 4262.54 | 0.44 | 9589.17 |
| 4174 | 120x120 | 220x220 | 7 | 35 | 150 | 238 | 242 | 0.07 | 3864.16 | 0.43 | 8911.55 |
| ***Adult Control*** | | | | | | | | | | | |
| 2145 | 120x120 | 220x220 | 8 | 35 | 150 | 198 | 370 | 0.05 | 6621.83 | 0.44 | 15039.94 |
| 2315 | 120x120 | 220x220 | 6 | 35 | 150 | 192 | 395 | 0.05 | 7336.25 | 0.47 | 15742.92 |
| 2316 | 120x120 | 220x220 | 7 | 35 | 150 | 213 | 374 | 0.05 | 6389.06 | 0.52 | 12315.31 |
| 1820 | 120x120 | 220x220 | 6 | 35 | 150 | 177 | 407 | 0.05 | 7394.84 | 0.45 | 16332.84 |
| 1821 | 120x120 | 220x220 | 7 | 35 | 150 | 188 | 403 | 0.05 | 6803.28 | 0.45 | 15153.02 |
| ***Old Control*** | | | | | | | | | | | |
| 6952 | 120x120 | 220x220 | 7 | 35 | 150 | 194 | 512 | 0.05 | 8067.56 | 0.46 | 17687.58 |
| 6953 | 120x120 | 220x220 | 7 | 35 | 150 | 184 | 442 | 0.05 | 6909.73 | 0.42 | 16363.18 |
| 6954 | 120x120 | 220x220 | 7 | 35 | 150 | 206 | 611 | 0.04 | 10987.67 | 0.52 | 21170.03 |
| 6957 | 120x120 | 220x220 | 8 | 35 | 150 | 192 | 488 | 0.05 | 7694.62 | 0.47 | 16438.07 |
| 6959 | 120x120 | 220x220 | 6 | 35 | 150 | 180 | 587 | 0.04 | 8139.73 | 0.46 | 17788.42 |
| ***Young GFAP-IL6*** | | | | | | | | | | | |
| 3424 | 120x120 | 220x220 | 7 | 35 | 150 | 180 | 284 | 0.06 | 3569.33 | 0.44 | 8145.72 |
| 3430 | 120x120 | 220x220 | 7 | 35 | 150 | 212 | 327 | 0.06 | 4101.70 | 0.49 | 8322.79 |
| 3432 | 120x120 | 220x220 | 7 | 35 | 150 | 202 | 303 | 0.06 | 3798.42 | 0.47 | 8060.09 |
| 3435 | 120x120 | 220x220 | 6 | 35 | 150 | 184 | 330 | 0.06 | 4061.35 | 0.47 | 8680.86 |
| 4175 | 120x120 | 220x220 | 7 | 35 | 150 | 195 | 311 | 0.06 | 3882.70 | 0.47 | 8302.37 |
| ***Adult GFAP-IL6*** | | | | | | | | | | | |
| 2330 | 120x120 | 220x220 | 7 | 35 | 150 | 186 | 516 | 0.04 | 8384.46 | 0.44 | 19147.54 |
| 2332 | 120x120 | 220x220 | 7 | 35 | 150 | 183 | 740 | 0.04 | 10690.88 | 0.42 | 25254.57 |
| 2334 | 120x120 | 220x220 | 7 | 35 | 150 | 158 | 534 | 0.04 | 8871.98 | 0.36 | 24445.13 |
| 2337 | 120x120 | 220x220 | 7 | 35 | 150 | 177 | 534 | 0.04 | 10086.99 | 0.41 | 24311.80 |
| 2338 | 120x120 | 220x220 | 7 | 35 | 150 | 156 | 451 | 0.05 | 7915.20 | 0.34 | 23041.35 |
| ***Old GFAP-IL6*** | | | | | | | | | | | |
| 7860 | 220x220 | 120x120 | 7 | 35 | 150 | 163 | 775 | 0.04 | 12771.21 | 0.37 | 34411.46 |
| 1287 | 220x220 | 120x120 | 6 | 35 | 150 | 145 | 526 | 0.04 | 10537.95 | 0.35 | 30192.45 |
| 1424 | 220x220 | 120x120 | 7 | 35 | 150 | 160 | 753 | 0.04 | 13607.05 | 0.37 | 36911.44 |
| 1426 | 220x220 | 120x120 | 7 | 35 | 150 | 142 | 661 | 0.04 | 10965.34 | 0.31 | 34847.61 |
| 1429 | 220x220 | 120x120 | 7 | 35 | 150 | 166 | 709 | 0.04 | 11798.10 | 0.36 | 33099.63 |

Table 2. Summary of stereological parameters used to count ChAT^+^ cholinergic cells in the medial septum and septal volume results.

| **Data set** | **Counting frame size (xy) µm^2^** | **Grid size (xy) µm^2^** | **Number of sections** | **Dissector height (µm)** | **Distance between sections (µm)** | **Number of sampling sites** | **Number of ChAT^+^ cells counted** | **Gunderson coefficient error (m=1)** | **Estimated number of ChAT^+^ cells** | **Measured Volume (mm³)** | **Density n/mm^3^** |
| --- | --- | --- | --- | --- | --- | --- | --- | --- | --- | --- | --- |
| ***Young Control*** | | | | | | | | | | | |
| 3433 | 120x120 | 220x220 | 7 | 35 | 150 | 96 | 238 | 0.07 | 3686.11 | 0.44 | 8292.41 |
| 3513 | 120x120 | 220x220 | 7 | 35 | 150 | 86 | 198 | 0.07 | 3273.99 | 0.40 | 8199.88 |
| 3993 | 120x120 | 220x220 | 7 | 35 | 150 | 87 | 262 | 0.06 | 3963.13 | 0.45 | 8883.05 |
| 3994 | 120x120 | 220x220 | 7 | 35 | 150 | 96 | 238 | 0.07 | 3686.11 | 0.44 | 8292.41 |
| 4174 | 120x120 | 220x220 | 7 | 35 | 150 | 91 | 235 | 0.07 | 3652.34 | 0.43 | 8416.94 |
| ***Adult Control*** | | | | | | | | | | | |
| 2145 | 120x120 | 220x220 | 8 | 35 | 150 | 198 | 199 | 0.07 | 3561.47 | 0.44 | 8089.05 |
| 2315 | 120x120 | 220x220 | 6 | 35 | 150 | 192 | 226 | 0.07 | 4197.45 | 0.47 | 9007.35 |
| 2316 | 120x120 | 220x220 | 7 | 35 | 150 | 213 | 226 | 0.07 | 3860.77 | 0.52 | 7441.87 |
| 1820 | 120x120 | 220x220 | 6 | 35 | 150 | 177 | 279 | 0.06 | 5069.19 | 0.45 | 11196.22 |
| 1821 | 120x120 | 220x220 | 7 | 35 | 150 | 188 | 332 | 0.06 | 5604.69 | 0.45 | 12483.38 |
| ***Old Control*** | | | | | | | | | | | |
| 6952 | 120x120 | 220x220 | 7 | 35 | 150 | 194 | 228 | 0.07 | 3592.58 | 0.46 | 7876.49 |
| 6953 | 120x120 | 220x220 | 7 | 35 | 150 | 184 | 234 | 0.07 | 3658.09 | 0.42 | 8662.86 |
| 6954 | 120x120 | 220x220 | 7 | 35 | 150 | 206 | 258 | 0.06 | 4639.64 | 0.52 | 8939.23 |
| 6957 | 120x120 | 220x220 | 8 | 35 | 150 | 192 | 226 | 0.07 | 3563.49 | 0.47 | 7612.71 |
| 6959 | 120x120 | 220x220 | 6 | 35 | 150 | 180 | 276 | 0.06 | 3827.20 | 0.46 | 8363.89 |
| ***Young GFAP-IL6*** | | | | | | | | | | | |
| 3424 | 120x120 | 220x220 | 7 | 35 | 150 | 44 | 239 | 0.06 | 3003.77 | 0.44 | 6855.03 |
| 3430 | 120x120 | 220x220 | 7 | 35 | 150 | 44 | 211 | 0.07 | 2646.66 | 0.49 | 5370.36 |
| 3432 | 120x120 | 220x220 | 7 | 35 | 150 | 44 | 195 | 0.07 | 2444.53 | 0.47 | 5187.19 |
| 3435 | 120x120 | 220x220 | 6 | 35 | 150 | 44 | 254 | 0.06 | 3126.01 | 0.47 | 6681.64 |
| 4175 | 120x120 | 220x220 | 7 | 35 | 150 | 44 | 225 | 0.07 | 2805.24 | 0.47 | 6023.56 |
| ***Adult GFAP-IL6*** | | | | | | | | | | | |
| 2330 | 120x120 | 220x220 | 7 | 35 | 150 | 186 | 178 | 0.08 | 2892.31 | 0.44 | 6605.15 |
| 2332 | 120x120 | 220x220 | 7 | 35 | 150 | 183 | 215 | 0.07 | 3106.13 | 0.42 | 7337.47 |
| 2334 | 120x120 | 220x220 | 7 | 35 | 150 | 158 | 180 | 0.08 | 2990.56 | 0.36 | 8239.94 |
| 2337 | 120x120 | 220x220 | 7 | 35 | 150 | 177 | 192 | 0.07 | 3626.78 | 0.41 | 8741.31 |
| 2338 | 120x120 | 220x220 | 7 | 35 | 150 | 156 | 153 | 0.08 | 2685.20 | 0.34 | 7816.69 |
| ***Old GFAP-IL6*** | | | | | | | | | | | |
| 7860 | 220x220 | 120x120 | 7 | 35 | 150 | 163 | 167 | 0.08 | 2751.99 | 0.37 | 7415.11 |
| 1287 | 220x220 | 120x120 | 6 | 35 | 150 | 145 | 146 | 0.08 | 2924.98 | 0.35 | 8380.41 |
| 1424 | 220x220 | 120x120 | 7 | 35 | 150 | 160 | 153 | 0.08 | 2764.78 | 0.37 | 7499.94 |
| 1426 | 220x220 | 120x120 | 7 | 35 | 150 | 142 | 146 | 0.08 | 2422.00 | 0.31 | 7697.06 |
| 1429 | 220x220 | 120x120 | 7 | 35 | 150 | 166 | 161 | 0.08 | 2679.12 | 0.36 | 7516.29 |

Table 3. Summary of stereological data for Iba-1^+^ microglia and ChAT^+^ cholinergic cells in the mouse medial septum.

| **Animal cohort** | **Animals per cohort (N)** | **Estimated number of Iba-1^+^ cells (n)** | **Estimated Iba-1^+^ cell density (n/mm^3^)** | **Estimated number of ChAT^+^ cells (n)** | **Estimated ChAT^+^ cell density (n/mm^3^)** | **Medial septal volume (mm^3^)** |
| --- | --- | --- | --- | --- | --- | --- |
| ***Control*** | | | | | | |
| Young | 5 | 3835.97 ± 147.86 | 8862.45 ± 385.19 | 3652.34 ± 110.00 | 8416.94 ± 121.53 | 0.43 ± 0.01 |
| Adult | 5 | 6909.05 ± 197.81 | 14916.81 ± 690.16 | 4458.71 ± 381.89 | 9643.57 ± 952.47 | 0.47 ± 0.01 |
| Old | 5 | 8359.86 ± 692.27 | 17889.46 ± 873.19 | 3856.20 ± 201.13 | 8291.04 ± 244.51 | 0.47 ± 0.02 |
| ***GFAP-IL6*** | | | | | | |
| Young | 5 | 3882.70 ± 96.22 | 8302.37 ± 106.52 | 2805.24 ± 121.98 | 6023.56 ± 335.45 | 0.47 ± 0.01 |
| Adult | 5 | 9189.90 ± 521.17 | 23240.08 ± 1082.76 | 3060.20 ± 157.60 | 7748.11 ± 367.97 | 0.39 ± 0.02 |
| Old | 5 | 11935.93 ± 565.94 | 33892.52 ± 1109.38 | 2708.57 ± 82.12 | 7701.76 ± 175.77 | 0.35 ± 0.01 |

Table 4. Summary of morphological analyses results for medial septal Iba-1^+^ microglia.

| **Animal cohort** | **Number of cells (N)** | **Soma area (µm)^2^** | **Soma perimeter (µm)** | **Soma circularity** | **Processes from Soma (n)** | **Total length of processes (µm)** | **Nodes (n)** | **Convex 3D surface area (µm)^2^** | **Convex 3D volume (µm)^3^** | **Convex perimeter (µm)** |
| --- | --- | --- | --- | --- | --- | --- | --- | --- | --- | --- |
| ***Control/ChAT-eGFP*** | | | | | | | | | | |
| Young | 30 | 25.37 ± 0.77 | 19.68 ± 0.34 | 0.80 ± 0.01 | 4.80 ± 0.22 | 626.93 ± 34.19 | 47.50 ± 3.19 | 6574.06 ±279.82 | 31116.46 ± 2273.01 | 199.16 ± 5.11 |
| Adult | 30 | 25.07 ± 0.73 | 19.46 ± 0.31 | 0.81 ± 0.01 | 4.93 ± 0.20 | 630.39 ± 21.76 | 50.00 ± 2.34 | 5911.36 ±250.50 | 30508.69 ± 1912.81 | 179.59 ± 4.72 |
| Old | 30 | 35.05 ± 1.35 | 23.45 ± 0.50 | 0.78 ± 0.01 | 4.67 ± 0.23 | 376.01 ± 20.30 | 26.97 ± 1.95 | 3765.12 ±175.14 | 15524.22 ± 1084.64 | 143.61 ± 4.15 |
| ***GFAP-IL6*** | | | | | | | | | | |
| Young | 30 | 30.62 ± 1.15 | 22.08 ± 0.53 | 0.77 ± 0.01 | 4.73 ± 0.22 | 363.01 ± 14.45 | 24.77 ± 1.14 | 4304.68 ± 207.46 | 14586.78 ± 893.02 | 167.75 ± 4.84 |
| Adult | 30 | 32.82 ± 0.80 | 23.13 ± 0.34 | 0.76 ± 0.01 | 4.70 ± 0.23 | 383.37 ± 17.62 | 31.13 ± 1.97 | 3512.83 ± 127.23 | 14290.09 ± 839.92 | 134.16 ± 2.58 |
| Old | 30 | 41.50 ± 1.36 | 26.95 ± 0.58 | 0.72 ± 0.01 | 4.43 ± 0.23 | 240.36 ± 12.70 | 16.10 ± 1.29 | 2938.77 ± 139.31 | 10837.01 ± 839.25 | 126.31 ± 3.69 |

Table 5. Summary of sholl analyses, area under the curve (AUC), results for medial septal Iba-1^+^ microglia.

| **Animal cohort** | **Number of data points (N)** | **Intersections (n)** | **Length (µm)** | **Surface area (µm)^2^** | **Volume (µm)^3^** | **Average diameter (µm)** | **Nodes (n)** |
| --- | --- | --- | --- | --- | --- | --- | --- |
| ***Control*** | | | | | | | |
| Young | 405 | 73.23 ± 7.82 | 626.80 ± 63.94 | 1269.00 ± 126.40 | 221.80 ± 22.78 | 6.49 ± 0.48 | 47.50 ± 6.87 |
| Adult | 405 | 75.27 ± 7.10 | 630.40 ± 53.94 | 1283.00 ± 106.40 | 233.50 ± 22.54 | 6.08 ± 0.41 | 50.00 ± 6.79 |
| Old | 405 | 46.60 ± 6.11 | 376.00 ± 46.50 | 835.00 ± 107.50 | 166.80 ± 25.22 | 5.22 ± 0.49 | 26.97 ± 5.01 |
| ***GFAP-IL6*** | | | | | | | |
| Young | 405 | 45.93 ± 5.53 | 362.80 ± 39.91 | 745.00 ± 75.83 | 134.10 ± 14.32 | 5.77 ± 0.45 | 24.77 ± 4.34 |
| Adult | 405 | 47.03 ± 6.39 | 383.40 ± 45.24 | 948.70 ± 111.30 | 212.30 ± 29.88 | 5.66 ± 0.42 | 31.13 ± 5.44 |
| Old | 405 | 30.53 ± 4.08 | 240.40 ± 31.52 | 602.70 ± 82.26 | 136.10 ± 22.14 | 5.56 ± 0.56 | 16.10 ± 3.60 |

Table 6. Summary of morphological analyses results for medial septal ChAT^+^ cholinergic cells.

| **Animal cohort** | **Number of cells (N)** | **Soma area (µm)^2^** | **Soma perimeter (µm)** | **Soma circularity** | **Processes from Soma (n)** | **Total length of dendrites (µm)** | **Nodes (n)** | **Convex 3D surface area (µm)^2^** | **Convex 3D volume (µm)^3^** | **Convex perimeter (µm)** |
| --- | --- | --- | --- | --- | --- | --- | --- | --- | --- | --- |
| ***Control*** | | | | | | | | | | |
| Young | 25 | 112.90 ± 4.54 | 44.69 ± 1.13 | 0.68 ± 0.01 | 3.28 ± 0.18 | 488.63 ± 24.25 | 4.56 ± 0.37 | 16732.84 ± 1093.35 | 86863.08 ± 9211.57 | 407.56 ± 7.36 |
| Adult | 25 | 137.68 ± 8.41 | 48.30 ± 1.70 | 0.72 ± 0.01 | 3.48 ± 0.22 | 292.83 ± 20.40 | 1.88 ± 0.33 | 14957.24 ± 1335.71 | 83589.76 ± 12180.93 | 371.78 ± 15.60 |
| Old | 25 | 107.72 ± 5.70 | 43.29 ± 1.28 | 0.70 ± 0.01 | 2.92 ± 0.21 | 196.32 ± 24.03 | 1.88 ± 0.28 | 8816.82 ± 1540.48 | 44820.80 ± 11650.09 | 263.10 ± 18.84 |
| ***GFAP-IL6*** | | | | | | | | | | |
| Young | 25 | 136.83 ± 5.86 | 48.26 ± 1.26 | 0.68 ± 0.01 | 3.40 ± 0.16 | 320.89 ± 17.76 | 3.40 ± 0.37 | 11039.00 ± 700.90 | 47944.75 ± 4909.54 | 326.45 ± 11.62 |
| Adult | 25 | 124.87 ± 5.34 | 45.49 ± 1.20 | 0.74 ± 0.01 | 2.40 ± 0.13 | 148.46 ± 9.93 | 1.36 ± 0.22 | 6350.51 ± 529.89 | 27422.15 ± 3412.91 | 231.69 ± 13.43 |
| Old | 25 | 134.05 ± 9.07 | 46.80 ± 1.72 | 0.73 ± 0.01 | 2.40 ± 0.22 | 133.99 ± 9.58 | 1.28 ± 0.20 | 5549.70 ± 441.91 | 20121.16 ± 2599.33 | 201.78 ± 12.08 |

Table 7. Summary of sholl analyses, area under the curve (AUC), results for medial septal ChAT^+^ cholinergic cells.

| **Animal cohort** | **Number of data points (N)** | **Intersections (n)** | **Length (µm)** | **Surface area (µm)^2^** | **Volume (µm)^3^** | **Average diameter (µm)** | **Nodes (n)** |
| --- | --- | --- | --- | --- | --- | --- | --- |
| ***Control*** | | | | | | | |
| Young | 431 | 33.28 ± 2.93 | 488.60 ± 43.41 | 1110.00 ± 101.80 | 235.90 ± 28.05 | 8.12 ± 0.64 | 4.56 ± 1.33 |
| Adult | 431 | 24.24 ± 2.66 | 292.60 ± 32.26 | 872.60 ± 105.30 | 252.40 ± 42.67 | 9.60 ± 0.96 | 1.88 ± 0.98 |
| Old | 431 | 16.40 ± 3.08 | 196.30 ± 36.22 | 598.10 ± 98.84 | 172.30 ± 32.72 | 7.64 ± 0.99 | 1.88 ± 0.98 |
| ***GFAP-IL6*** | | | | | | | |
| Young | 431 | 23.04 ± 2.82 | 320.90 ± 40.24 | 760.50 ± 87.31 | 172.10 ± 24.10 | 7.59 ± 0.70 | 3.40 ± 1.39 |
| Adult | 431 | 11.92 ± 1.86 | 148.50 ± 20.28 | 466.50 ± 64.76 | 135.10 ± 22.27 | 7.17 ± 0.85 | 1.36 ± 0.81 |
| Old | 431 | 11.12 ± 1.76 | 134.00 ± 19.24 | 449.70 ± 67.26 | 143.80 ± 33.79 | 7.34 ± 1.06 | 1.28 ± 0.76 |

Table 8. Summary of hippocampal pyramidal neuronal spine density analysis.

| **Animal cohort** | **Number of samples (N)** | **Spine density (1/µm)** |
| --- | --- | --- |
| ***Control*** |  |  |
| Young | 24 | 1.23 ± 0.05 |
| Adult | 24 | 1.30 ± 0.05 |
| Old | 24 | 1.10 ± 0.06 |
| ***GFAP-IL6*** |  |  |
| Young | 24 | 0.98 ± 0.04 |
| Adult | 24 | 1.02 ± 0.04 |
| Old | 24 | 0.66 ± 0.03 |
